# Supplementary material for: Outcomes after cancer diagnosis in children and adult patients with congenital heart disease in Sweden: a registry-based cohort study
Source: BMJ Open. 2024 Apr 17;14(4):e083237. doi: 10.1136/bmjopen-2023-083237 (PMC11029300; doi:10.1136/bmjopen-2023-083237)

Supplementary tables and figures

Supplementary Table 1. Congenital heart disease diagnosis according to the International Statistical Classification of Diseases and Related Health Problems

Diagnosis	ICD-8	ICD-9*	ICD-10
Common arterial trunk	746,09	745A	Q200
Transposition of the great arteries	746,19	745B	Q203
Tetralogy of Fallot	746,29	745C	Q213
Ventricular septal defect	746,39	745E	Q210
Atrial septal defect/patent foramen ovale	746,42	745F	Q211
Congenital tricuspid stenosis or atresia	746,54	746B	Q224
Ebstein’s anomaly	746,54	746C	Q225
Congenital stenosis of the aortic valve	746,73	746D	Q230
Congenital insufficiency of the aortic valve	746,79	746E	Q231
Congenital mitral stenosis	746,59	746F	Q232
Congenital mitral insufficiency	746,59	746G	Q233
Hypoplastic left heart syndrome	746,74	746H	Q234
Congenital subaortic stenosis	746,79	746W	Q244
Cor triatriatum	746,89	746W	Q242
Infundibular pulmonic stenosis	746,63	746W	Q243
Congenital coronary artery anomalies	747,69	746W	Q245
Congenital heart block	746,89	746W	Q246
Coarctation of the aorta	747,19	747B	Q251
Interruption of the aortic arch	747,19	747B	Q252<br>Q253
Other unspecified congenital malformations of the aorta	747,29	747C	Q254<br>Q258<br>Q259
Congenital malformations of the pulmonary artery	747,34<br>747,39	747D	Q255<br>Q256<br>Q257
Congenital malformations of the great veins	747,49<br>747,59	747E	Q260<br>Q261<br>Q262<br>Q263<br>Q264
Cor biloculare	746,89	745H	Q208
Double outlet right ventricle	746,19	745B	Q201
Double outlet left ventricle	746,19	745B	Q202
Double inlet ventricle	746,37	745D	Q204
Congenitally corrected transposition/discordant atrioventricular and ventriculoatrial connection	746,19	745B	Q205

Isomerism of atrial appendages	746,89	745W	Q206
Unspecified congenital malformations of the cardiac chambers	746,89	746X	Q208<br>Q209
Atrioventricular septal defect	746,47<br>746,46<br>746,43	745G	Q212
Aortopulmonary septum defect	746,09	745A	Q214
Other congenital malformations of the cardiac septum	746,89	745W	Q218
Unspecified congenital malformations of the cardiac septum	746,99	745X	Q219
Pulmonary valve atresia	746,64	746A	Q220
Congenital stenosis of the pulmonary valve	746,63	746A	Q221
Congenital pulmonary valve insufficiency	746,69	746A	Q222
Other congenital malformations of the pulmonary valve	746,69	746A	Q223
Hypoplastic right heart syndrome	746,69	746B	Q226
Other congenital malformations of the tricuspid valve	746,54	746B	Q228<br>Q229
Other congenital malformations of aortic and mitral valves	746,89	746W	Q238<br>Q239
Other specified congenital malformations of the heart	746,89	746W	Q248
Unspecified congenital malformations of the heart	746,99	746X	Q249
Patent ductus arteriosus	747,09	747A	Q250

ICD, International Classification of Diseases. *Swedish version of the ICD-9.

Supplementary Table 2. Classification of CHD

		CHD diagnosis	ICD-8	ICD-9	ICD-10
**Complex lesions**	**Lesion group 1:** conotruncal defects	Common arterial trunk	746.09	745A	Q200
		Aortopulmonary septum defect	746.09	745A	Q214
		Double outlet right ventricle	746.19	745B	Q201
		Double outlet left ventricle	746.19	745B	Q202
		Transposition of great vessels	746.19	745B	Q203
		Discordant atrioventricular connection (ccTGA)		745B	Q205
		Tetralogy of Fallot	746.29	745C	Q213
	**Lesion group 2:** severe nonconotruncal defects	Endocardial cushion defects	746.43<br>746.46<br>746.47	745G	Q212
		Common ventricle	746.37	745D	Q204
		Hypoplastic left heart syndrome	746.74	745H	Q234
**Non-complex lesions**	**Lesion group 3:** coarcatio of the aorta	Coarctation of the aorta	747.19	747B	Q251
	**Lesion group 4:** Ventricular septal defect	Ventricular septal defect	746.39	745E	Q210
	**Lesion group 5:** Atrial septal defect	Atrial septal defect	746.42	745F	Q211
	**Lesion group 6:** Other heart and circulatory system anomalies	All other congenital heart disease diagnoses that are not included in the above five lesion groups			

Supplementary Table 3. Cancer diagnosis according to the International Statistical Classification of Diseases and Related Health Problems

Diagnosis	ICD-8	ICD-9*	ICD-10
Malignant neoplasms of lip, oral cavity and pharynx	140-149	140-149	C00-C14
Malignant neoplasms of digestive organs	150-159	150-159	C15-C26
Malignant neoplasms of respiratory and intrathoracic organs	160-163	160-165	C30-C39
Malignant neoplasms of bone and articular cartilage	170-171	170	C40-C41
Melanoma and other malignant neoplasms of skin	172-173	172-173	C43-C44
Malignant neoplasms of mesothelial and soft tissue	-	158,162,163, 171, 173	C45-C49
Malignant neoplasms of breast	174	174-175	C50
Malignant neoplasms of female genital organs	180-184	179-184,236C	C51-C58
Malignant neoplasms of male genital organs	185-187	185-187	C60-C63
Malignant neoplasms of urinary tract	188-189	188-189	C64-C68
Malignant neoplasms of eye, brain and other parts of central nervous system	190-192	190-192	C69-C72
Malignant neoplasms of thyroid and other endocrine glands	193-194	193-194	C73-C75
Malignant neoplasms o ill-defined, secondary and unspecified sites	195-198	195-199	C76-C80
Malignant neoplasms, stated or presumed to be primary, of lymphoid, haematopoietic and related tissue	200-209	200-209	C81-C96
Malignant neoplasms of independent (primary) multiple sites	199	199	C97

ICD, International Classification of Diseases. *Swedish version of the ICD-9.

Supplementary Table 4. Syndromes diagnosis according to the International Statistical Classification of Diseases and Related Health Problems

Diagnosis	ICD8	ICD9	ICD10
Down syndrome	7593	758A	Q90
Noonan syndrome	-	-	Q87.1
Di Georges syndrome (22q11-deletion)	-	279L	D82.1
Klinefelter	759,51	758H	Q98
Turners	759,50	758G	Q96
Williams-Beuren	-	-	Q93.8
Edwards (trisomi 18)	759,40	758C	Q91
Patau (trisomi 13)	759,41	758B	Q91
CFC (cardiofaciocutaneous syndrome, Costellos syndrome, CHARGE, Smith-Magenis	-	-	Q87.8
Neurofibromatosis type 1	743,40	237H	Q85.0
Constitutional aplastic anaemia (incl Fanconis anaemia)	284	284A	D61.0
Heterotaxia (situs inversus)	759.0	759D	Q89.3

Supplementary table 5. Comorbidity diagnoses according to the International Statistical Classification of Diseases and Related Health Problems

Diagnosis	ICD8	ICD9	ICD10
Hypertension	400-404	401-405	I10-I15
Diabetes mellitus	250	250	E10-E14
Heart failure	427.00	428	I50
Myocardial infarction	410	410	I21
Atrial fibrillation	427.92	427D	I48
Ischemic stroke	433, 434	434, 436	I63-I64

Supplementary table 6. Follow-up time

Follow-up time	CHD	Controls
All cancer patients		
Mean, years (SD)	**9.2±9.3***	**7.8±7.8***
Median, years (IQR)	6.3 (1.9-14.5)	5.3 (2.0-11.3)
Patients with cancer who died		
Mean, years (SD)	3.3±6.6	2.9±4.9
Median, years (IQR)	**0.8 (0.2-3.3)***	**1.3 (0.7-3.0)***
Patients with cancer who died (excl. syndromes and transplant recipients)		
Mean, years (SD)	3.7±7.1	2.8±4.6
Median, years (IQR)	1.0 (0.3-3.6)	1.2 (0.7-2.9)

Numbers in bold denotes significance (p <0.05)

Supplementary table 7. Cancer diagnosis in patients with CHD and controls who died (all patients)

Cancer diagnosis	Cases	Controls
Malignant neoplasms, stated or presumed to be primary, of lymphoid, haematopoietic and related tissue	59 (28.5%)	117 (15.4%)
Malignant neoplasms of ill-defined, secondary and unspecified sites	43 (20.8%)	198 (26.0%)
Malignant neoplasms of eye, brain and other parts of central nervous system	26 (12.6%)	147 (19.3%)
Malignant neoplasms of digestive organs	17 (8.2%)	46 (6.0%)
Malignant neoplasms of mesothelial and soft tissue	13 (6.3%)	59 (7.7%)
Malignant neoplasms of urinary tract	<10	20 (2.6%)
Malignant neoplasms of thyroid and other endocrine glands	<10	30 (3.9%)
Malignant neoplasms of lip, oral cavity and pharynx	<10	11 (1.4%)
Malignant neoplasms of breast	<10	28 (3.7%)
Malignant neoplasms of female genital organs	<10	23 (3.0%)
Malignant neoplasms of respiratory and intrathoracic organs	<10	17 (2.2%)
Malignant neoplasms of bone and articular cartilage	<5	30 (3.9%)
Melanoma and other malignant neoplasms of skin	<5	24 (3.1%)
Malignant neoplasms of male genital organs	<5	10 (1.3%)
Malignant neoplasms of independent (primary) multiple sites	0	<5

Supplementary table 8. Cancer diagnosis in patients with CHD and controls who died (excluding syndromes and transplant recipients)

Cancer diagnosis	Cases	Controls
Malignant neoplasms of ill-defined, secondary and unspecified sites	40 (27.0%)	193 (26.7%)
Malignant neoplasms of eye, brain and other parts of central nervous system	22 (14.9%)	141 (19.4%)
Malignant neoplasms, stated or presumed to be primary, of lymphoid, haematopoietic and related tissue	21 (14.2%)	109 (15.0%)
Malignant neoplasms of digestive organs	12 (8.1%)	45 (6.2%)
Malignant neoplasms of mesothelial and soft tissue	12 (8.1%)	51 (7.0%)
Malignant neoplasms of thyroid and other endocrine glands	<10	29 (4.0%)
Malignant neoplasms of breast	<10	27 (3.7%)
Malignant neoplasms of female genital organs	<10	23 (3.2%)
Malignant neoplasms of lip, oral cavity and pharynx	<10	11 (1.5%)
Malignant neoplasms of respiratory and intrathoracic organs	<10	16 (2.2%)
Malignant neoplasms of urinary tract	<5	20 (2.8%)
Malignant neoplasms of bone and articular cartilage	<5	29 (4.0%)
Malignant neoplasms of male genital organs	<5	<10
Melanoma and other malignant neoplasms of skin	<5	20 (2.8%)
Malignant neoplasms of independent (primary) multiple sites	0	<5

Supplementary table 9. Deaths

Deaths	CHD	Controls
Female/Male	69/70	213/214
Birth period (patients with cancer)		
- 1970-1979	41	183
- 1980-1989	36	89
- 1990-1999	34	82
- 2000-2009	14	60
- 2010-2017	14	13
Lesion group (patients with cancer)		
- Complex lesions	35 (29.2%)	
- Non-complex lesions	104 (16.3%)	

Supplementary table 10. Mortality IR divided by gender, lesion groups and birth cohorts

Mortality IR*	CHD (incl/excl syndromes+Tx)	Controls (incl/excl syndromes+Tx)
In total cancer population	1.99 / 1.72	1.49 / 1.46
Cancer population divided by:		
- ***Gender***		
Male	2.03 / 1.82	1.56 / 1.51
Female	1.95 / 1.61	1.43 / 1.41
- ***Lesion groups***		
Complex lesions	3.70 / 2.40	
Non-complex lesions	1.72 / 1.63	
- ***Birth period***		
1970-1979	1.89 / 1.78	1.46 / 1.41
1980-1989	2.05 / 1.50	1.24 / 1.19
1990-1999	1.78 / 1.43	1.60 / 1.63
2000-2009	1.52 / 1.23	1.86 / 1.90
2010-2017	6.13 / 6.88	2.54 / 2.01

* per 100 person-years

Supplementary table 11. Mortality HR divided by gender, lesion groups and birth cohorts

Mortality hazard ratio	HR (95% CI)	
Divided by gender	Incl. syndromes and tpx	Excl. syndromes and tpx
Unadjusted model	Gender 1 **1.48 (1.13-1.93)**<br>Gender 2 **1.62 (1.23-2.13)**	Gender 1 **1.36 (1.00-1.87)**<br>Gender 2 1.33 (0.95-1.86)
Model 1 (adjusted for birth period and age at cancer diagnosis)	Gender 1 **1.36 (1.04-1.79)**<br>Gender 2 **1.36 (1.03-1.80)**	Gender 1 1.27 (0.92-1.74)<br>Gender 2 1.14 (0.81-1.60)
Model 2 (adjusted for birth period, age at cancer diagnosis and comorbidities)	Gender 1 1.32 (0.99-1.74)<br>Gender 2 1.18 (0.88-1.59)	Gender 1 1.24 (0.90-1.72)<br>Gender 2 1.09 (0.77-1.54)
Divided by lesion groups	Incl. syndromes and tpx	Excl. syndromes and tpx
Unadjusted model	Complex **2.96 (1.97-4.46)**<br>Non-complex **1.33 (1.07-1.65)**	Complex **1.99 (1.12-3.55)**<br>Non-complex 1.27 (0.99-1.64)
Model 1 (adjusted for birth period, age at cancer diagnosis and gender)	Complex **2.64 (1.72-4.07)**<br>Non-complex **1.17 (0.94-1.46)**	Complex 1.76 (0.98-3.17)<br>Non-complex 1.14 (0.89-1.47)
Model 2 (adjusted for birth period, age at cancer diagnosis, gender and comorbidities)	Complex **2.32 (1.45-3.72)**<br>Non-complex 1.11 (0.88-1.39)	Complex 1.70 (0.90-3.19)<br>Non-complex 1.11 (0.86-1.44)
Divided by birth cohorts	Incl. syndromes and tpx	Excl. syndromes and tpx
Unadjusted model	1970-1989 **1.71 (1.33-2.21)**<br>1990-2017 **1.36 (1.01-1.83)**	1970-1989 **1.49 (1.11-2.00)**<br>1990-2017 1.16 (0.80-1.68)
Model 1 (adjusted for age at cancer diagnosis and gender)	1970-1989 **1.50 (1.16-1.94)**<br>1990-2017 1.16 (0.86-1.57)	1970-1989 1.32 (0.98-1.77)<br>1990-2017 1.01 (0.70-1.47)
Model 2 (adjusted for age at cancer diagnosis, gender and comorbidities)	1970-1989 **1.37 (1.05-1.80)**<br>1990-2017 1.08 (0.80-1.48)	1970-1989 1.26 (0.93-1.71)<br>1990-2017 1.00 (0.69-1.46)

Supplementary table 12. Age at cancer diagnosis

**All patients**	**CHD**	**Controls**
Age 0-17 years at cancer diagnosis, n (%)	451 (59.5%)	1416 (38.6%)
Age 18+ years at cancer diagnosis, n (%)	307 (40.5%)	2254 (61.4%)
Mean, years (SD)	**16.5±14.5***	**22.7±14.2***
Median, years (IQR)	**13.0 (2.9–30.0)***	**24.6 (8.6–35.1)***
**All patients – excl. syndromes/Tx**	**CHD**	**Controls**
Age 0-17 years at cancer diagnosis, n (%)	299 (53.0%)	1345 (37.6%)
Age 18+ years at cancer diagnosis, n (%)	265 (47.0%)	2229 (62.4%)
Mean, years (SD)	**18.5±14.8***	**23.0±14.1***
Median, years (IQR)	**16.6 (3.9-32.2)**	**25.0 (9.1-35.2)***
**Patients who died**	**CHD**	**Controls**
Mean, years (SD)	**14.2±14.1***	**17.0±13.5***
Median, years (IQR)	**9.3 (1.7-24.0)***	**14.8 (3.8-29)***
**Patients who died – excl. syndromes/Tx**	**CHD**	**Controls**
Mean, years (SD)	16.2±15.0	17.2±13.6
Median, years (IQR)	13.5 (1.6-29.9)	15.1 (3.9-29.1)

CHD=congenital heart disease. Tx= transplant recipients. SD=standard deviation. IQR=interquartile range.
Numbers in bold denotes significance (p <0.05)

Supplementary table 13. Age at death in patients with cancer

**Age at death – all patients**	**CHD**	**Controls**
Mean, years (SD)	17.5±14.8	19.8±13.9
Median, years (IQR)	**15.1 (3.6-30.7)***	**18.5 (6.1-32.7)***
**Age at death – excl. syndromes/Tx**	**CHD**	**Controls**
Mean, years (SD)	19.8±15.5	20.0±13.9
Median, years (IQR)	19.5 (4.7-35.6)	18.7 (6.1-32.7)

CHD=congenital heart disease. Tx= transplant recipients. SD=standard deviation. IQR=interquartile range.
Numbers in bold denotes significance (p <0.05)

Supplementary figure 1. Kaplan-Meier survival

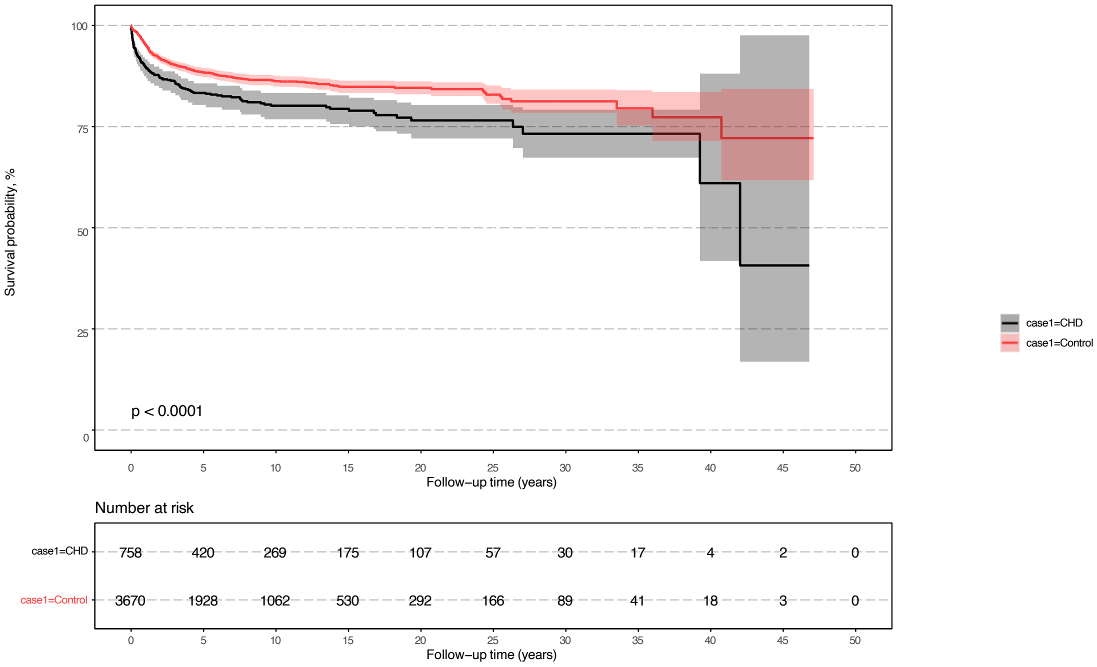

Supplementary figure 2a. Cause of death patients with CHD and cancer: complex lesions

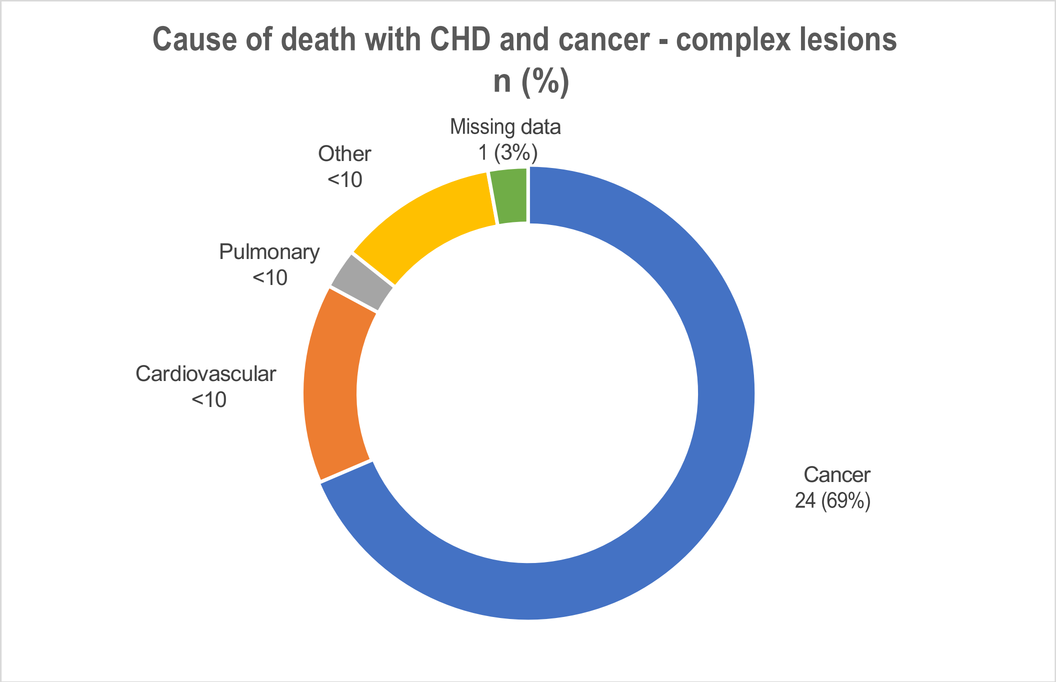

* All cause of deaths from patients and controls from the year 2017 were missing, because of later registration.

Supplementary figure 2b. Cause of death patients with CHD and cancer: non-complex lesions

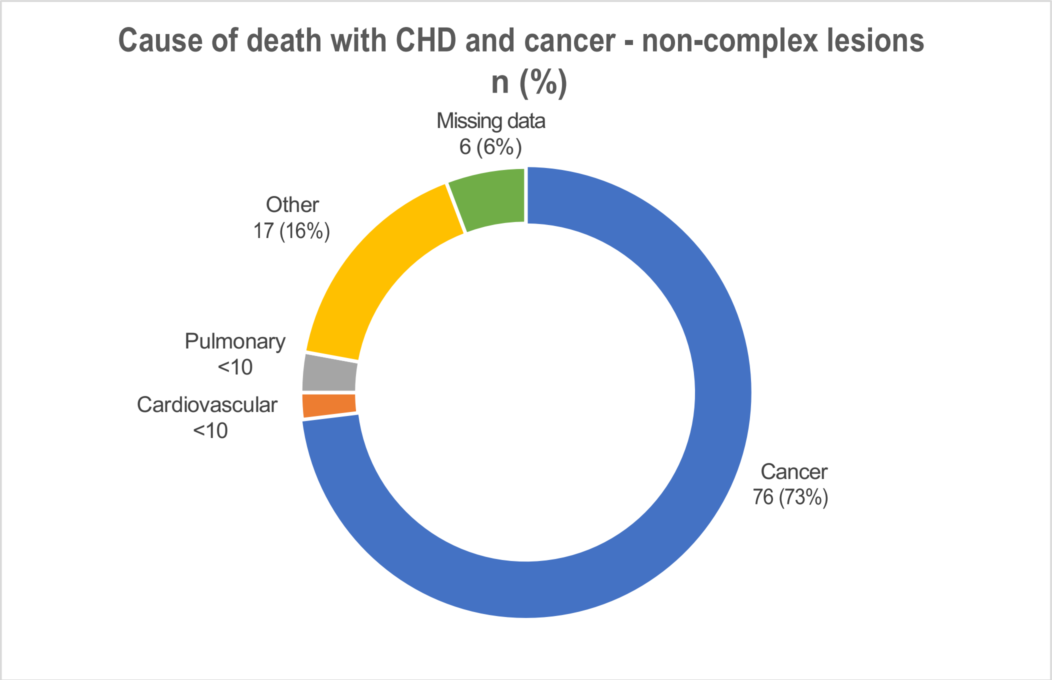

* All cause of deaths from patients and controls from the year 2017 were missing, because of later registration.

Supplementary figure 3a. Age at death in patients with CHD and cancer cause-of-death (all patients)

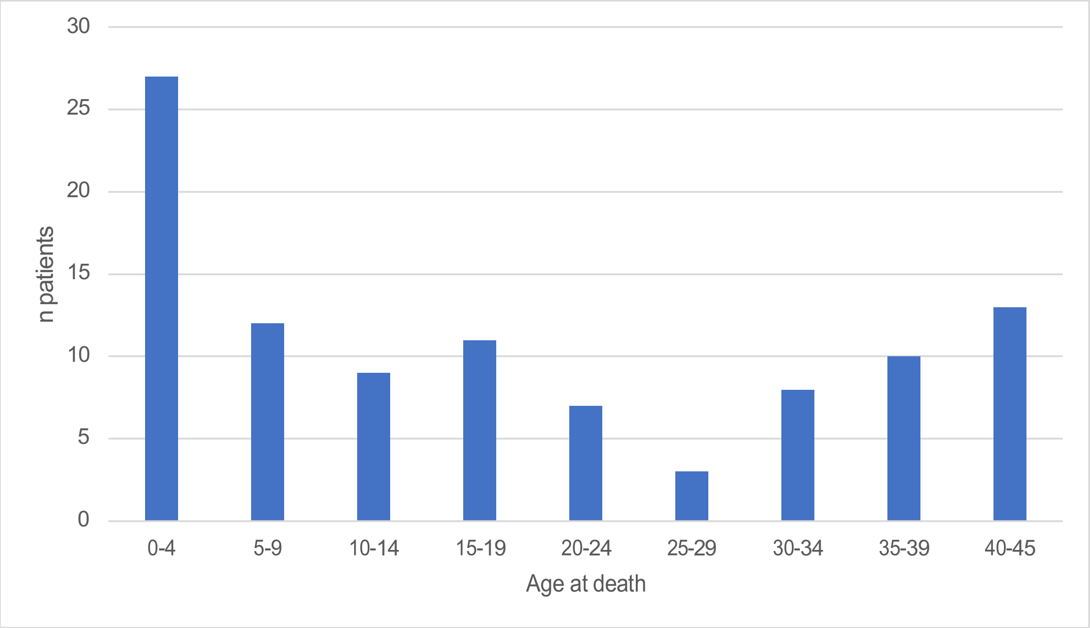

Supplementary figure 3b. Age at death in patients with CHD and cancer cause-of-death (excl. syndromes and transplant recipients)

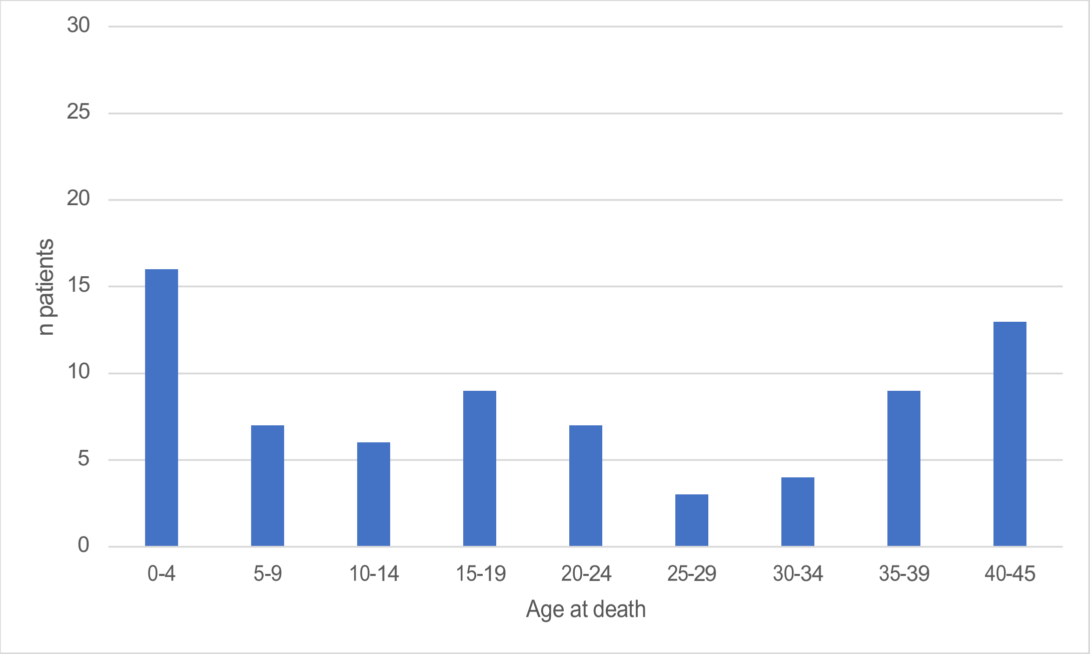

Supplement: Supplementary data [file bmjopen-2023-083237supp001.pdf]
