# Supplementary material for: Outcomes after cancer diagnosis in children and adult patients with congenital heart disease in Sweden: a registry-based cohort study
Source: BMJ Open. 2024 Apr 17;14(4):e083237. doi: 10.1136/bmjopen-2023-083237 (PMC11029300; doi:10.1136/bmjopen-2023-083237)
Supplement: Author's manuscript [file bmjopen-2023-083237.draft_revisions.pdf]

BMJ Open is committed to open peer review. As part of this commitment we make the peer review history of every article we publish publicly available.

When an article is published we post the peer reviewers' comments and the authors' responses online. We also post the versions of the paper that were used during peer review. These are the versions that the peer review comments apply to.

The versions of the paper that follow are the versions that were submitted during the peer review process. They are not the versions of record or the final published versions. They should not be cited or distributed as the published version of this manuscript.

BMJ Open is an open access journal and the full, final, typeset and author-corrected version of record of the manuscript is available on our site with no access controls, subscription charges or pay-per-view fees (http://bmjopen.bmj.com).

If you have any questions on BMJ Open's open peer review process please email info.bmjopen@bmj.com

BMJ Open

Outcomes after cancer diagnosis in children and adult patients with congenital heart disease

Journal:	*BMJ Open*
Manuscript ID	bmjopen-2023-083237
Article Type:	Original research
Date Submitted by the Author:	14-Dec-2023
Complete List of Authors:	Karazisi, Christina; University of Gothenburg, Department of Molecular and Clinical Medicine; Sahlgrenska University Hospital, Department of Medicine, Geriatrics and Emergency Medicine, Region Västra Götaland<br>Dellborg, Mikael; University of Gothenburg, Department of Molecular and Clinical Medicine; Sahlgrenska University Hospital, Department of Medicine, Geriatrics and Emergency Medicine, Region Västra Götaland<br>Mellgren, Karin; Sahlgrenska University Hospital, Department of Pediatric Oncology<br>Giang, Kok Wai; University of Gothenburg, Department of Molecular and Clinical Medicine; Sahlgrenska University Hospital, Department of Medicine, Geriatrics and Emergency Medicine, Region Västra Götaland<br>Skoglund, Kristofer; University of Gothenburg, Department of Molecular and Clinical Medicine<br>Eriksson, Peter; University of Gothenburg, Department of Molecular and Clinical Medicine; Sahlgrenska University Hospital, Department of Medicine, Geriatrics and Emergency Medicine, Region Västra Götaland<br>mandalenakis, zacharias; University of Gothenburg, Department of Molecular and Clinical Medicine; Sahlgrenska University Hospital, Department of Medicine, Geriatrics and Emergency Medicine, Region Västra Götaland
Keywords:	Epidemiology < TROPICAL MEDICINE, Congenital heart disease < CARDIOLOGY, CARDIOLOGY, ONCOLOGY, Prognosis

SCHOLARONE™
Manuscripts

I, the Submitting Author has the right to grant and does grant on behalf of all authors of the Work (as defined in the below author licence), an exclusive licence and/or a non-exclusive licence for contributions from authors who are: i) UK Crown employees; ii) where BMJ has agreed a CC-BY licence shall apply, and/or iii) in accordance with the terms applicable for US Federal Government officers or employees acting as part of their official duties; on a worldwide, perpetual, irrevocable, royalty-free basis to BMJ Publishing Group Ltd ("BMJ") its licensees and where the relevant Journal is co-owned by BMJ to the co-owners of the Journal, to publish the Work in this journal and any other BMJ products and to exploit all rights, as set out in our licence.

The Submitting Author accepts and understands that any supply made under these terms is made by BMJ to the Submitting Author unless you are acting as an employee on behalf of your employer or a postgraduate student of an affiliated institution which is paying any applicable article publishing charge ("APC") for Open Access articles. Where the Submitting Author wishes to make the Work available on an Open Access basis (and intends to pay the relevant APC), the terms of reuse of such Open Access shall be governed by a Creative Commons licence – details of these licences and which Creative Commons licence will apply to this Work are set out in our licence referred to above.

Other than as permitted in any relevant BMJ Author's Self Archiving Policies, I confirm this Work has not been accepted for publication elsewhere, is not being considered for publication elsewhere and does not duplicate material already published. I confirm all authors consent to publication of this Work and authorise the granting of this licence.

For peer review only

Outcomes after cancer diagnosis in children and adult patients with congenital heart disease

Christina Karazisi, MD,[1,2]; Mikael Dellborg, MD,[1,2,3]; Karin Mellgren, MD,[4]; Kok Wai Giang, PhD,[1,2]; Kristofer Skoglund, MD,[1]; Peter Eriksson, MD,[1,2,3]; Zacharias Mandalenakis, MD[1,2,3]

[1] Institute of Medicine, Department of Molecular and Clinical Medicine, Sahlgrenska Academy, University of Gothenburg, Sweden
[2] Department of Medicine, Geriatrics and Emergency Medicine, Region Västra Götaland, Sahlgrenska University Hospital/Östra, Gothenburg, Sweden
[3] Adult Congenital Heart Disease Unit, Sahlgrenska University Hospital, Gothenburg, Sweden
[4] Department of Pediatric Oncology, the Queen Silvia Children's Hospital, Sahlgrenska University Hospital, Gothenburg, Sweden

Corresponding author:
Christina Karazisi, MD
Department of Molecular and Clinical Medicine
Sahlgrenska University Hospital
Diagnosvägen 11, SE-416 50 Gothenburg, Sweden
E-mail: christina.karazisi@vgregion.se
Tel: +4631 342000; Fax: +4631 191416

Manuscript word count: 3433 (not including title, abstract, references)

Keywords: congenital heart disease; cardio-oncology; cancer mortality; prognosis

For peer review only - http://bmjopen.bmj.com/site/about/guidelines.xhtml

Abstract

Objective: Patients with congenital heart disease (CHD) have an increased cancer risk. The aim of this study is to determine cancer-related mortality in CHD patients compared with non-CHD controls, compare ages at cancer diagnosis and death, and explore the most fatal cancer diagnoses.

Methods and analysis: CHD patients born between 1970 and 2017 were identified using Swedish Health Registers. Each was matched by birth year and sex with 10 non-CHD controls. Included were those born in Sweden with a cancer diagnosis.

Results: Cancer developed in 758 out of 67814 CHD patients (1.1%), with 139 deaths (18.3%). Cancer was the cause in 71.9% of these cases. Across all CHD patients, cancer accounted for 1.8% of deaths. Excluding patients with syndromes and transplant recipients, mortality risk between CHD patients with cancer and controls showed no significant difference (HR 1.17; 95% CI 0.93–1.49). CHD patients had a lower median age at cancer diagnosis—13.0 years (IQR 2.9–30.0) in CHD vs. 24.6 years (IQR 8.6–35.1) in controls. Median age at death was 15.1 years (IQR 3.6–30.7) in CHD patients vs. 18.5 years (IQR 6.1–32.7) in controls. The top three fatal cancer diagnoses were CNS tumors, hematologic, and digestive organ malignancies.

Conclusions: Cancer-related deaths constituted 1.8% of all mortalities across all CHD patients. Among CHD patients with cancer, 18.3% died, with cancer being the cause in 71.9% of cases. Although CHD patients have an increased cancer risk, their mortality risk post-diagnosis does not significantly differ from non-CHD patients.

For peer review only - http://bmjopen.bmj.com/site/about/guidelines.xhtml

Strengths and limitations of this study

- The primary strength of this study lies in its nationwide scope; it includes all patients born in Sweden between 1970 and 2017 who has been diagnosed with CHD.
- The follow-up is from birth, in a country with universal health coverage.
- A major limitation is that it relies solely on register-based data, which excludes clinical details. Consequently, we lacked specific information regarding the cancer treatment administered to patients.
- Moreover, we did not have information on cancer stages.

Key messages

What is already known on this topic: Patients with congenital heart disease (CHD) have an increased risk of cancer compared to controls without CHD – however, studies in outcomes after cancer diagnosis in these patients are sparse.

What this study adds: Patients with CHD develop cancer at a younger age compared with controls without CHD. The three leading causes of cancer-related fatalities were CNS tumors, hematologic cancers, and malignancies of the digestive organs in both CHD and non-CHD patients. The mortality risk in CHD patients with cancer was not significantly different from that in cancer patients without CHD, after excluding patients with syndromes and transplant recipients.

How this study might affect research, practice or policy: Further studies are needed to evaluate the vulnerability of patients with syndromes and concurrent cancer, as they constituted almost 30% of total facilities.

For peer review only - http://bmjopen.bmj.com/site/about/guidelines.xhtml

Introduction

Nearly 2% of children are born with a congenital heart defect.[1] These defects range from simple and uncomplicated, sometimes resolving with age, to extremely complex conditions necessitating multiple surgeries and/or catheter interventions for survival. The prognosis for congenital heart disease (CHD) has improved significantly, with over 97% now expected to reach adulthood.[2]

In previous studies, we have found that the overall cancer risk for CHD patients in Sweden is higher than in matched controls without CHD. Notably, this risk in children and young adults is more than double that of the matched controls.[3, 4] As age increases, so does the risk of acquired diseases such as cancer. Furthermore, evidence suggests that CHD patients are less likely to participate in screenings for malignancies, including cervical, breast, and colon cancer.[5, 6]

Cancer ranks as the fourth leading cause of death among adult CHD patients, preceded by heart failure, pneumonia, and sudden cardiac death.[7] Venkatesh et al. recently reported on malignancy outcomes in a single-center cohort comprising 68 adults with CHD and cancer. In this group, 24% of the patients died during a median follow-up period of five years, with cancer being the cause of death in 69% of these cases.[8] However, to our knowledge, there has been no studies exploring whether there is an elevated risk of mortality in CHD patients with cancer compared with cancer patients without CHD. Furthermore, there are no studies on long-term outcomes for children with CHD following a cancer diagnosis.

In this study, we aim to investigate the prevalence of cancer-related mortality in pediatric and adult patients with CHD born in Sweden between 1970 and 2017. We also examine whether

For peer review only - http://bmjopen.bmj.com/site/about/guidelines.xhtml
For peer review only

there is an increased risk of mortality in patients with CHD who also have cancer, compared with a control group of cancer patients without CHD. In addition, we will investigate whether the age at cancer diagnosis and mortality differs between patients with CHD and those without CHD. Furthermore, we will identify the most common fatal cancer diagnoses in this population.

Methods

Study population and design

This nationwide registry-based cohort study utilized data from the Swedish National Inpatient Register (established in 1964, encompassing all cardiothoracic centers since 1970, and achieving national coverage in 1987), the Swedish National Outpatient Register (complete since 2001) and the Swedish Cause of Death Register (complete since 1968). The International Statistical Classification of Diseases and Related Health Problems (ICD) facilitated the identification of diagnoses.

Patients diagnosed with CHD born between 1970 and 2017 were identified for the study. Each CHD patient was paired with 10 controls *without* CHD, matched by sex and birth year, sourced from the Swedish Total Population Register. Patients not born in Sweden were excluded. The study included 67814 and 583709 controls; among these, we identified individuals diagnosed with cancer (758 CHD patients and 3670 controls) for inclusion. The Swedish National Board of Health and Welfare and Statistics Sweden provided case-control identification and facilitated register linkage. In the final dataset, all national registration numbers were replaced with a code, ensuring data anonymization. Data analysis was conducted from January 2023 to October 2023.

For peer review only - http://bmjopen.bmj.com/site/about/guidelines.xhtml

This study was approved by the Gothenburg Regional Research Ethics Board (Gbg 912-16, T 619-18). The tenets of the Declaration of Helsinki were followed, and the need for patient consent was waived as the study utilized anonymized register-based data. Patients or the public were not involved in the design, or conduct, or reporting, or dissemination plans of our research. This report follows the Strengthening the Reporting of Observational Studies in Epidemiology (STROBE) reporting guidelines.

Definitions

CHD was defined using ICD codes, as outlined in Supplementary Table 1. The diagnoses of CHD were classified in accordance with the hierarchical CHD classification system initially described by Botto et al. and later modified by Liu et al.[9, 10] Patients were divided into two groups: those with complex lesions (lesion group 1–2) and those with non-complex lesions (lesion group 3–6) (Supplementary Table 2). Cancer was defined using ICD codes, as specified in Supplementary Table 3. Syndromes were defined using ICD codes, detailed in Supplementary Table 4. Comorbidities were defined using ICD codes according to Supplementary Table 5, prior to the date of cancer diagnosis.

Statistical analysis

All statistical analyses were performed using R v3.5.2 (R Foundation for Statistical Computing, Vienna, Austria). Baseline characteristics of patients are described with categorical data shown as numbers and percentages, while continuous data are presented as mean ± standard deviation (SD) or median and interquartile range (IQR). Descriptive statistics were used to report cancer-related mortality among all patients with CHD, expressed as numbers and percentages. The patients were observed from cancer diagnosis until death or the

For peer review only

study's conclusion (December 31, 2017). The median follow-up duration was 6.3 years (IQR 1.9–14.5) for the patients with CHD, and 5.3 years (IQR 2.0–11.3) for the controls. Mortality incidence rates (IR) were reported per 100 person-years, calculated as the total deaths divided by the cumulative follow-up time of the study population. Hazard ratios (HR) with 95% CIs were derived using Cox proportional hazard regression models. In these models, the control group served as the reference. The analysis was initially conducted without adjustments, followed by two adjusted models. The first adjusted model accounted for variables such as birth year, age at cancer diagnosis, and gender. The second adjusted model included the variables from the first model and additionally adjusted for comorbidities, namely hypertension, diabetes, myocardial infarction, ischemic stroke, atrial fibrillation, and heart failure. A *p*-value of less than 0.05 was considered statistically significant.

Results

Baseline characteristics

We identified 67814 patients born in Sweden between 1970 and 2017 who had been diagnosed with CHD, along with 583,709 matched controls without CHD who were matched by birth year and sex. Among these, 758 (1.1%) CHD patients and 3670 (0.6%) controls subsequently developed cancer. The characteristics of our study population are shown in Table 1.

The median follow-up duration post-cancer diagnosis was 6.3 years (IQR 1.9–14.5) for CHD patients and 5.3 years (IQR 2.0–11.3) for the control group (Supplementary Table 6).

Cancer diagnoses

Figure 1a–b shows the distribution of cancer diagnoses among CHD patients and controls. Among patients who died, the three predominant types diagnosed in those with CHD were

For peer review only - http://bmjopen.bmj.com/site/about/guidelines.xhtml

hematologic (36.7%), CNS tumors (16.5%), and digestive organ malignancies (11.5%). In controls, CNS tumors were the most common (20.4%), followed by hematologic malignancies (19.0%) and digestive organ malignancies (10.8%) (Supplementary Table 7). Upon excluding individuals with syndromes and those who had undergone transplants, CNS tumors emerged as the leading cancer diagnosis in CHD patients, followed by hematologic cancer and digestive organ malignancies, a trend consistent in both groups (Supplementary Table 8).

For peer review only

Mortality after cancer diagnosis

Among the patients studied, 139 patients (18.3%) with concurrent CHD and cancer died during the study period compared with 427 (11.6%) of the control group, who had cancer but not CHD (refer to Table 2 and Supplementary Table 9 for additional details). Kaplan–Meier survival analysis for patients with cancer, categorized by the presence or absence of CHD, is presented in Supplementary Figure 1. Among the 139 deceased patients, 35 (25.2%) had complex lesions, whereas 104 (74.8%) had non-complex lesions. Consequently, the mortality rate was 29.2% among patients with complex lesions and cancer, compared with 16.3% for patients with cancer having non-complex lesions.

Patients with CHD and cancer exhibited a higher mortality IR compared with controls diagnosed with cancer but without CHD (Supplementary Table 10). Notably, the most pronounced differences in mortality IRs were observed in the youngest birth cohort, spanning 2010–2017, in which the IR for CHD patients was 6.13, in contrast to 2.54 for controls.

The initial unadjusted analysis showed a higher mortality risk following cancer diagnosis in patients with CHD compared with controls without CHD (HR 1.55; 95% CI 1.28–1.88). This

For peer review only - http://bmjopen.bmj.com/site/about/guidelines.xhtml

increased risk persisted, albeit to a lesser extent, after excluding patients with syndromes and transplant recipients (HR 1.35; 95% CI 1.08–1.7) (Table 3). Subsequent analyses, adjusting for birth period, age at cancer diagnosis, and gender in the first model, and additionally for comorbidities in the second model, revealed that the increased risk remained significant (model 1: HR 1.38; 95% CI 1.13–1.68; model 2: HR 1.27; 95% CI 1.04–1.55). However, upon the exclusion of patients with syndromes and transplant recipients, the difference in mortality risk between patients with CHD and the control group was not statistically significant (model 1: HR 1.21; 95% CI 0.96–1.53; model 2: HR 1.17; 95% CI 0.93–1.49) (Table 3, Supplementary Table 11).

Cause of death

Cancer accounted for 1.8% of mortality cases in the overall CHD cohort, while it represented 72% of deaths among patients with both CHD and cancer (Table 2). In fewer than 10 instances among CHD patients with cancer, the cause of death was cardiovascular. Causes of death categorized by complex lesions and non-complex lesions are presented in Supplementary Figure 2a–b. Among patients with cancer as the cause of death, 26% (n = 26) also had a syndrome diagnosis. All cause of deaths from patients and controls from the year 2017 were missing, because of later registration. Therefore, seven patients who died 2017 had missing data of cause of death.

Age at cancer diagnosis

The analysis revealed a notable difference in the age at which cancer was diagnosed between patients with CHD and the control group. Specifically, the median age for cancer diagnosis in patients with CHD was 13.0 years (IQR 2.9–30.0), whereas it was 24.6 years (IQR 8.6–35.1) in the control group (Supplementary table 12). This difference persisted even after the

For peer review only - http://bmjopen.bmj.com/site/about/guidelines.xhtml

exclusion of patients with syndromes and transplant recipients. However, among patients with cancer who died, the initial observed difference in the age at cancer diagnosis between CHD patients and controls was no longer evident after the exclusion of patients with syndromes and transplant recipients (Supplementary Table 12).

Age at death

The median age at death was lower in patients with CHD and cancer—15.1 years (IQR 3.6–30.7) compared with 18.5 years (IQR 6.1–32.7) in controls with cancer. Excluding patients with syndromes and transplant recipients, the difference in age at death between CHD patients and controls was minimal, with a median age of 19.5 years (IQR 4.7–35.6) for CHD patients versus 18.7 years (IQR 6.1–32.7) for controls (Supplementary Table 13). Supplementary Figure 3a–b illustrates the distribution of age at death among patients with CHD and cancer.

Discussion

This study, to the best of our knowledge, represents the first to explore long-term outcomes following a cancer diagnosis in individuals with CHD, ranging in age from birth to a maximum of 47 years. Our analysis revealed that cancer developed in 758 (1.1%) of the patients with CHD and in 3670 (0.6%) of the controls. In previous studies, we demonstrated that the most common cancer types in CHD patients were largely similar to those in the control group, with the exclusion of patients with syndromes and transplant recipients.[4] In this study, the three most frequent cancer diagnoses for both CHD patients and controls were hematologic, skin, and CNS tumors. The three leading causes of cancer-related fatalities were CNS tumors, hematologic cancers, and malignancies of the digestive organs.

For peer review only - http://bmjopen.bmj.com/site/about/guidelines.xhtml

For peer review only

In a nationwide cohort study conducted in Taiwan, Lee et al. identified an elevated risk of various cancers, particularly hematologic, CNS, and head and neck malignancies, in children and adults with CHD.[11] Venkatesh et al., focusing on adults with CHD who developed cancer, reported the most frequent cancer types as hematologic, breast, skin, hepatocellular, and urologic cancers.[8] Notably, in that study, five out of the seven patients with hepatocellular carcinoma (HCC) had previously undergone Fontan palliation, a procedure associated with an increased HCC risk.[12] In our study, we categorized HCC under digestive organ malignancies, which emerged as one of the three most fatal cancers. However, fewer than five CHD patients who succumbed to their illnesses had HCC. This lower incidence in our study might be attributed to the younger age of our patients, considering that HCC risk notably increases over 30 years post-Fontan operation.[12]

During the study, 139 patients with CHD and cancer died, constituting 18.3% of the study population. Among these, cancer was the primary cause of death for 72% of patients with CHD. This finding aligns with the research by Venkatesh et al. on adult CHD patients, which reported a 24% mortality rate among cancer patients over a median follow-up period of 5 years post-cancer diagnosis, with cancer accounting for 69% of these deaths.[8]

In our total CHD cohort, 1.8% of all deaths were attributed to cancer. Previous studies have reported higher frequencies of cancer-related mortality in CHD patients, ranging from 6%–16%.[7, 13, 14] However, these studies focused exclusively on adult CHD patients. As age increases, the risk of acquiring other diseases also rises, potentially affecting mortality risk. For instance, in the study reporting 16% cancer-caused mortality, 42% of the patients had heart failure.[14] Conversely, in our CHD and cancer cohort, only 6.1% of patients had heart failure.

For peer review only - http://bmjopen.bmj.com/site/about/guidelines.xhtml

A recent study investigating causes of death in infants, children, and young adults up to 20 years old with CHD found that the primary cause of mortality in infants was CHD, followed by lung disease and infection. In children, the leading causes were CHD, neurologic disease, and infection.[15] The absence of cancer in this report likely reflects its relatively low incidence of cancer-related death in pediatric patients compared with adults. Our study, encompassing both children and adult patients, suggests that the generally lower frequency of cancer-related deaths in children might contribute to the reduced frequency of cancer-caused mortality observed in our cohort.

The mortality IRs were notably higher in CHD patients with cancer than in controls with cancer but without CHD. Overall, patients with CHD have an increased mortality risk relative to controls.[16] The highest mortality IR was found in the youngest birth cohort of patients with cancer.

In our analysis of whether patients *with* CHD *and* cancer face a heightened mortality risk compared with controls *without CHD* but *with* cancer, we found an increased risk in patients with CHD when factors such as birth period, age at cancer diagnosis, gender, and comorbidities were accounted for (HR 1.27; 95% CI 1.04–1.55). However, this increased risk was not significant upon excluding patients with syndromes and transplant recipients from the analysis.

Mortality rates among patients with CHD have declined over time, especially for those born after 1975.[16] Our study includes patients born after 1970 who were diagnosed with cancer. Increased survival rates into adulthood may have altered both the risk of cancer development

For peer review only - http://bmjopen.bmj.com/site/about/guidelines.xhtml
For peer review only

and the effectiveness of cancer treatment over time. This could explain the lack of a significant difference in survival rates between CHD patients and controls.

In Sweden, excluding malignant melanoma and basal cell carcinoma, skin cancer ranks as the second most common cancer diagnosis. Malignant melanoma is the fifth most common.[17] Our study did not differentiate between skin cancer types; all were categorized under a single group. Skin cancer incidence was higher in controls with cancer but without CHD, compared with CHD patients with cancer. However, it was not a common diagnosis among patients who died. This finding underscores the absence of increased mortality in CHD patients, given the higher incidence of this diagnosis in controls.

In the observed cohort, syndromes accounted for 41 deaths of the total 139 fatalities (29%), of which 26 were attributed to cancer (representing 26% of all cancer-related deaths among CHD patients). Among these 26 cancer-related deaths, 11 occurred in patients with complex lesions (accounting for 46% of cancer-related deaths in this subgroup) and 15 in those with non-complex lesions (constituting 20% of the deaths in this category). Previous studies have shown that children and adolescents with non-Hodgkin lymphoma, especially those with pre-existing conditions such as cancer predisposition syndromes (e.g. Down syndrome), immunodeficiencies, genetic disorders (e.g. Williams-Beuren syndrome), and other non-classifiable conditions, exhibit lower survival rates compared with their counterparts without such pre-existing conditions.[18] Our findings corroborate the heightened vulnerability of patients with syndromes and concurrent cancer.

In examining the age at cancer diagnosis, it was observed that patients with CHD were diagnosed at a generally younger age than those without CHD. Differences were noted in the

For peer review only - http://bmjopen.bmj.com/site/about/guidelines.xhtml

age at diagnosis among patients that died, excluding those with syndromes and transplant recipients. Recognizing the heightened risk of cancer at younger ages is crucial for timely detection and treatment, ultimately enhancing survival outcomes.

Strengths and limitations

The primary strength of our study lies in its nationwide scope; it includes all patients born in Sweden between 1970 and 2017 who had been diagnosed with CHD. Additionally, the longitudinal tracking of patients from birth enhances the robustness of our findings.

However, our study is not without limitations. It relies solely on register-based data, which excludes clinical details. Consequently, we lacked specific information regarding the cancer treatment administered to patients. It is important to note that cancer treatment has evolved over time. Moreover, we did not have access to information on cancer stages. Another limitation is our focus on cancer mortality rates without considering the diverse types of cancer present in the study group. The heterogeneity in cancer types and their therapies significantly influence survival and mortality rates. Despite these variations, the cancer types in CHD patients and controls exhibited similar frequency patterns, lending a degree of reliability to our findings. Additionally, it is important to note that Sweden has a universal health coverage system. Consequently, these results may not be generalizable to patients with CHD in countries with different health care systems.

For peer review only

Conclusions

In conclusion, this study revealed that only 1.8% of deaths among Swedish patients with CHD, with or without cancer, were attributable to cancer. Among those with both CHD and cancer, 18.3% died within a median follow-up duration of 6.3 years (IQR 1.9–14.5). In the CHD patients who died, cancer was identified as the cause of death in 71.9% of cases. Notably, the mortality risk in CHD patients with cancer did not significantly exceed that of controls with cancer alone, once patients with syndromes and transplant recipients were excluded from the analysis. These findings indicate that while CHD patients face an increased risk of developing cancer and tend to develop cancer at a younger age, their mortality risk post-cancer diagnosis does not significantly differ from that of patients without CHD, again excluding those with syndromes and transplant recipients.

Acknowledgment: We thank Phoebe Chi, MD, from Edanz (https://www.edanz.com/ac), for editing a draft of this manuscript.

Author contribution: All authors have contributed significantly to the submitted work. CK, MD, KM, KWG, KS, PE and ZM contributed to the conception and design of the study. CK, ZM and KWG did the experimental design and the analyses. CK and ZM prepared the manuscript and verified the data. CK, MD, KM, KWG, KS, PE and ZM critically reviewed the manuscript and approved the final manuscript. All authors had full access to all the data in the study and had final responsibility for the decision to submit for publication.

Funding/Support: The study was funded by the Swedish state under an agreement between the Swedish Government and County Councils (Grant Number: ALFGBG-955256), the Swedish Research Council (Grant Number: 2019-00193), the Swedish Heart-Lung

For peer review only - http://bmjopen.bmj.com/site/about/guidelines.xhtml

Foundation (Grant Number: 20190724), and the Swedish Childhood Cancer Fund (Grant Number: SP2017-0012).

Conflict of Interest Disclosures: None reported.

For peer review only

References

1. Giang KW, Mandalenakis Z, Fedchenko M, et al. Congenital heart disease: changes in recorded birth prevalence and cardiac interventions over the past half-century in Sweden. *Eur J Prev Cardiol*. 2023;30(2):169-76.
2. Mandalenakis Z, Giang KW, Eriksson P, et al. Survival in children with congenital heart disease: Have we reached a peak at 97%? *J Am Heart Assoc*. 2020;9(22):e017704.
3. Mandalenakis Z, Karazisi C, Skoglund K, et al. Risk of cancer among children and young adults with congenital heart disease compared with healthy controls. *JAMA Netw Open.* 2019;2(7):e196762.
4. Karazisi C, Dellborg M, Mellgren K, et al. Risk of cancer in young and older patients with congenital heart disease and the excess risk of cancer by syndromes, organ transplantation and cardiac surgery: Swedish health registry study (1930-2017). *Lancet Reg Health Eur*. 2022;18:100407.
5. Blue GM, Kirk EP, Sholler GF, Harvey RP, Winlaw DS. Congenital heart disease: current knowledge about causes and inheritance. *Med J Aust*. 2012;197(3):155-9.
6. Christman MP, Castro-Zarraga M, Defaria Yeh D, Liberthson RR, Bhatt AB. Adequacy of cancer screening in adult women with congenital heart disease. *ISRN Cardiol*. 2013;2013:827696.
7. Diller GP, Kempny A, Alonso-Gonzalez R, et al. Survival prospects and circumstances of death in contemporary adult congenital heart disease patients under follow-up at a large tertiary centre. *Circulation*. 2015;132(22):2118-25.
8. Venkatesh P, Yan KL, Bravo-Jaimes K, Yang EH, Lluri G. Outcomes of malignancy in adults with congenital heart disease: a single center experience. *Cardiooncology*. 2022;8(1):20.
9. Botto LD, Lin AE, Riehle-Colarusso T, Malik S, Correa A. Seeking causes: Classifying and evaluating congenital heart defects in etiologic studies. *Birth Defects Res A Clin Mol Teratol*. 2007;79(10):714-27.
10. Liu S, Joseph KS, Lisonkova S, et al. Association between maternal chronic conditions and congenital heart defects: a population-based cohort study. *Circulation*. 2013;128(6):583-9.
11. Lee YS, Chen YT, Jeng MJ, et al. The risk of cancer in patients with congenital heart disease: a nationwide population-based cohort study in Taiwan. *PLoS One*. 2015;10(2):e0116844.
12. Ohuchi H, Hayama Y, Nakajima K, Kurosaki K, Shiraishi I, Nakai M. Incidence, predictors, and mortality in patients with liver cancer after Fontan operation. *J Am Heart Assoc*. 2021;10(4):e016617.
13. Goldstein SA, D'Ottavio A, Spears T, et al. Causes of death and cardiovascular comorbidities in adults with congenital heart disease. *J Am Heart Assoc*. 2020;9(14):e016400.
14. Van Bulck L, Goossens E, Morin L, et al. Last year of life of adults with congenital heart diseases: causes of death and patterns of care. *Eur Heart J.* 2022;43(42):4483-92.

For peer review only - http://bmjopen.bmj.com/site/about/guidelines.xhtml

15. Williams JL, Torok RD, D'Ottavio A, et al. Causes of death in infants and children with congenital heart disease. *Pediatr Cardiol*. 2021;42(6):1308-15.

16. Dellborg M, Giang KW, Eriksson P, et al. Adults with congenital heart disease: Trends in event-free survival past middle age. *Circulation*. 2023;147(12):930-8.

17. Cancerfonden. Cancer i siffror https://static-files.cancerfonden.se/Cancer-i-siffror-2023.pdf. Accessed 9 November 2023.

18. Attarbaschi A, Carraro E, Abla O, et al. Non-Hodgkin lymphoma and pre-existing conditions: spectrum, clinical characteristics and outcome in 213 children and adolescents. *Haematologica*. 2016;101(12):1581-91.

For peer review only

Tables 1-3

Table 1. Study population

Characteristic	CHD	Controls
***All patients*, No.**	67814	583709
Patients with cancer, No. (% of total)	758 (1.1%)	3670 (0.6%)
- Male	360 (47.5%)	1663 (45.3%)
- Female	398 (52.5%)	2007 (54.7%)
Birth period (patients with cancer), No. (%)		
- 1970–1979	222 (29.3%)	1538 (41.9%)
- 1980–1989	158 (20.8%)	832 (22.7%)
- 1990–1999	169 (22.3%)	616 (16.8%)
- 2000–2009	130 (17.2%)	489 (13.3%)
- 2010–2017	79 (10.4%)	195 (5.3%)
Lesion group (patients with cancer), No. (%)		
- Complex lesions	120 (15.8%)	
- Non-complex lesions	638 (84.2%)	
Comorbidities (patients with cancer), No. (%)		
- Hypertension	27 (3.6%)	44 (1.2%)
- Diabetes	12 (1.6%)	42 (1.1%)
- Myocardial infarction	1 (0.1%)	3 (0.1%)
- Ischemic stroke	8 (1.1%)	7 (0.2%)
- Atrial fibrillation	21 (2.8%)	10 (0.3%)
- Heart failure	46 (6.1%)	6 (0.2%)
Syndromes, n (%)	159 (21%)	75 (2.0%)
- **Complex**	42	61
- **Non-complex**	117	14
Transplant recipients, n (%)	36 (4.7%)	23 (0.6%)

For peer review only

Table 2. Deaths

**Total deaths**	**CHD**	**Controls**
In total population, No. (%)	5689 (0.08%)	4117 (0.007%)
In cancer patients (complex/non-complex), No.	139 (35/104)	427
% of deaths in cancer patients	139/758 = 18,3%	427/3670 = 11.6%
In patients with syndromes (complex/non-complex)	41 (15/26)	17
Transplant recipients	<10	<10
**Cancer cause of death***		
Total deaths in which cause of death was cancer, No.	100	370
Complex lesions	24	-
Non-complex lesions	76	-
In patients with syndromes (complex/non-complex)	26 (11/15)	15
Transplant recipients	<10	<5
Percentage of cancer-related death		
In total population deaths	100/5689 = 1,8%	370/4117 = 9,0%
In cancer patients	100/751 = 13,3%	370/3642 = 10,1%
In cancer patients deaths	100/139 = 71,9%	370/427 = 86,7%

*Excluding patients with missing causes of death.

Table 3. Mortality after cancer diagnosis

**Mortality hazard ratio**	**HR (95% CI)**	
All patients	Incl. syndromes and tpx	Excl. syndromes and tpx
Unadjusted model	**1.55 (1.28–1.88)***	**1.35 (1.08–1.70)***
Model 1 (adjusted for birth period, age at cancer diagnosis, and gender)	**1.38 (1.13–1.67)***	1.21 (0.96–1.53)
Model 2 (adjusted for birth period, age at cancer diagnosis, gender, and comorbidities)	**1.27 (1.03–1.55)***	1.17 (0.93–1.49)

****Numbers in bold denotes significance ($p < 0.05$).***
Abbreviation: tpx, transplant recipients

For peer review only

Figure 1a. Cancer diagnoses in patients with CHD

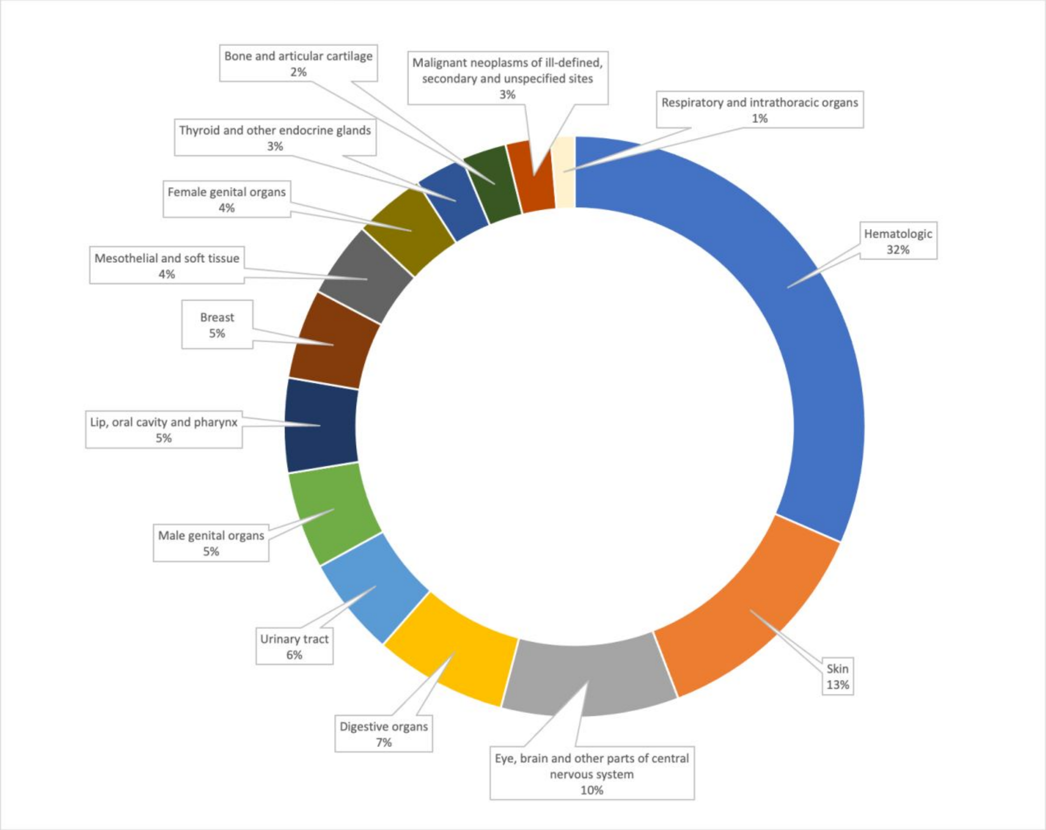

Figure 1b. Cancer diagnoses in controls without CHD

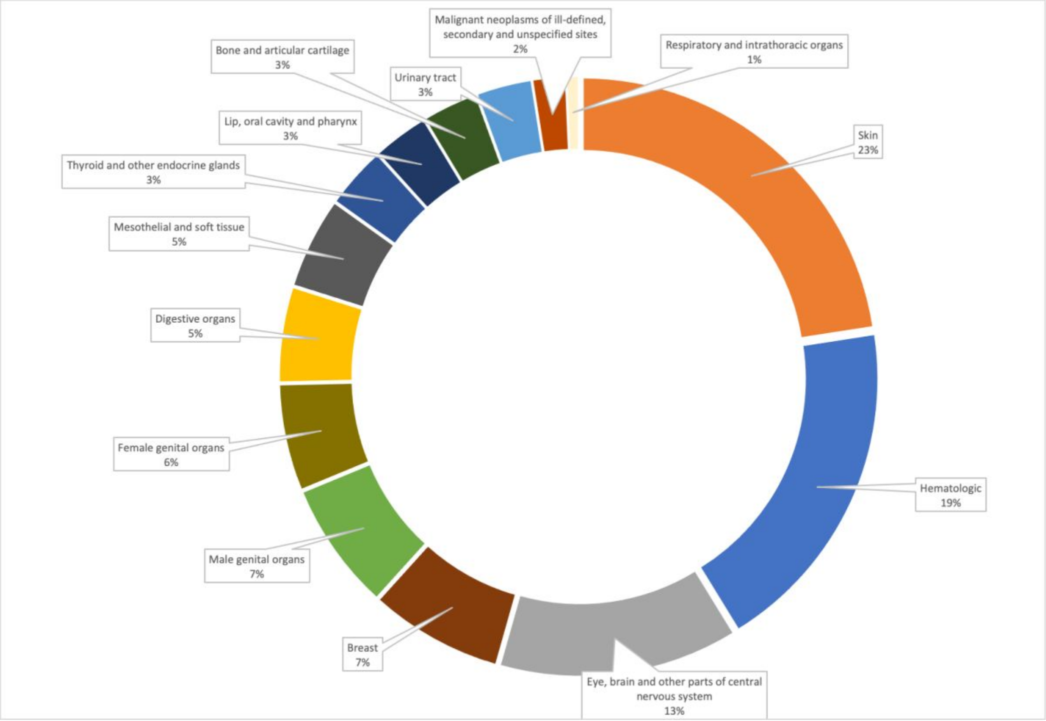

Supplementary tables and figures

Supplementary Table 1. Congenital heart disease diagnosis according to the International Statistical Classification of Diseases and Related Health Problems

Diagnosis	ICD-9*	ICD-10
Tetralogy of Fallot	745C	Q21.3
Transposition of the great vessels	745B	Q20.3
Common arterial trunk	745A	Q20.0
Ventricular septal defect	745E	Q21.0
Atrial septal defect or patent foramen ovale	745F	Q21.1
Congenital tricuspid stenosis or atresia	746B	Q22.4
Ebstein's anomaly	746C	Q22.5
Congenital stenosis of the aortic valve	746D	Q23.0
Congenital insufficiency of the aortic valve	746E	Q23.1
Congenital mitral stenosis	746F	Q23.2
Congenital mitral insufficiency	746G	Q23.3
Hypoplastic left heart syndrome	746H	Q23.4
Congenital subaortic stenosis	746W	Q24.4
Cor triatriatum	746W	Q24.2
Infundibular pulmonic stenosis	746W	Q24.3
Congenital coronary vessel anomalies	746W	Q24.5
Congenital heart block	746W	Q24.6
Coarctation of the aorta	747B	Q25.1
Interruption of the aortic arch (atresia or stenosis of the aorta)	747B	Q25.2, Q25.3
Other unspecified congenital malformations of the aorta	747C	Q25.4, Q25.8, Q25.9
Congenital malformations of the pulmonary artery	747D	Q25.5–Q25.7
Congenital malformations of the great veins	747E	Q26
Cor biloculare	745H	Q20.8
Double outlet right ventricle	745B	Q20.1
Double outlet left ventricle	745B	Q20.2
Double inlet ventricle	745D	Q20.4
Discordant atrioventricular connection	745B	Q20.5
Isomerism of atrial appendages	745W	Q20.6
Unspecified congenital malformations of the cardiac chambers	746X	Q20.8, Q20.9
Atrioventricular septal defect	745G	Q21.2
Aortopulmonary septum defect	745W	Q21.4
Other congenital malformations of the cardiac septum	745W	Q21.8
Unspecified congenital malformations of the cardiac septum	745X	Q21.9

For peer review only

Pulmonary valve atresia	746A	Q22.0
Congenital stenosis of the pulmonary valve	746A	Q22.1
Congenital pulmonary valve insufficiency	746A	Q22.2
Other congenital malformations of the pulmonary valve	746A	Q22.3
Hypoplastic right heart syndrome	746B	Q22.6
Other congenital malformations of the tricuspid valve	746B	Q22.8, Q22.9
Other congenital malformations of aortic and mitral valves	746W	Q23.8, Q23.9
Congenital phlebectasia	747G	Q27.4
Other specified congenital malformations of the heart	746W	Q24.8
Unspecified congenital malformations of the heart	746X	Q24.9
Patent ductus arteriosus	747A	Q25.0
Unspecified congenital malformations of the circulation	747X	Q28.9
Sequestration of the lungs	748F	Q33.2
Secondary hypertension	405	I15.8, I15.9
Vitium organicum cordis (VOC)	-	I33–37

ICD, International Classification of Diseases. *Swedish version of the ICD-9.

Supplementary Table 2. Classification of CHD

		CHD diagnosis	ICD-9	ICD-10
**Complex lesions**	**Lesion group 1:** conotruncal defects	Common arterial trunk	745A	Q200
		Aortopulmonary septum defect	745A	Q214
		Double outlet right ventricle	745B	Q201
		Double outlet left ventricle	745B	Q202
		Transposition of great vessels	745B	Q203
		Discordant atrioventricular connection (ccTGA)	745B	Q205
		Tetralogy of Fallot	745C	Q213
	**Lesion group 2:** severe nonconotruncal defects	Endocardial cushion defects	745G	Q212
		Common ventricle	745D	Q204
		Hypoplastic left heart syndrome	745H	Q234
**Non-complex lesions**	**Lesion group 3:** coarcatio of the aorta	Coarctation of the aorta	747B	Q251
	**Lesion group 4:** Ventricular septal defect	Ventricular septal defect	745E	Q210
	**Lesion group 5:** Atrial septal defect	Atrial septal defect	745F	Q211
	**Lesion group 6:** Other heart and circulatory system anomalies	All other congenital heart disease diagnoses that are not included in the above five lesion groups		

For peer review only

Supplementary Table 3. Cancer diagnosis according to the International Statistical Classification of Diseases and Related Health Problems

Diagnosis	ICD-9*	ICD-10
Malignant neoplasms of lip, oral cavity and pharynx	140-149	C00-C14
Malignant neoplasms of digestive organs	150-159	C15-C26
Malignant neoplasms of respiratory and intrathoracic organs	160-165	C30-C39
Malignant neoplasms of bone and articular cartilage	170	C40-C41
Melanoma and other malignant neoplasms of skin	172-173	C43-C44
Malignant neoplasms of mesothelial and soft tissue	158,162,163, 171, 173	C45-C49
Malignant neoplasms of breast	174-175	C50
Malignant neoplasms of female genital organs	179-184,236C	C51-C58
Malignant neoplasms of male genital organs	185-187	C60-C63
Malignant neoplasms of urinary tract	188-189	C64-C68
Malignant neoplasms of eye, brain and other parts of central nervous system	190-192	C69-C72
Malignant neoplasms of thyroid and other endocrine glands	193-194	C73-C75
Malignant neoplasms o ill-defined, secondary and unspecified sites	195-199	C76-C80
Malignant neoplasms, stated or presumed to be primary, of lymphoid, haematopoietic and related tissue	200-209	C81-C96
Malignant neoplasms of independent (primary) multiple sites	199	C97

ICD, International Classification of Diseases. *Swedish version of the ICD-9.

Supplementary Table 4. Syndromes diagnosis according to the International Statistical Classification of Diseases and Related Health Problems

Diagnosis	ICD9	ICD10
Downs syndrom	758A	Q90
Noonan		Q87.1
Di Georges syndrome (22q11-deletion)	279L	D82.1
Klinefelter	758H	Q98
Turners	758G	Q96
Williams-Buren		Q93.8
Edwards (trisomi 18)	758C	Q91
Patau (trisomi 13)	758B	Q91
CFC (cardio-facio-cutant syndrom, costellossyndrom, CHARGE, Smith-Magenis		Q87.8
Neurofibromatosis type 1	237H	Q85.0
Constitutional aplastic anemia (incl Fanconis anemia)	284A	D61.0
Heterotaxia (situs inversus)	759D	Q89.3

For peer review only

Supplementary table 5. Comorbidity diagnoses according to the International Statistical Classification of Diseases and Related Health Problems

Diagnosis	ICD9	ICD10
Hypertension	401-405	I10-I15
Diabetes mellitus	250	E10-E14
Heart failure	428	I50
Myocardial infarction	410	I21
Atrial fibrillation	427D	I48
Ischemic stroke	434, 436	I63-I64

Supplementary table 6. Follow-up time

Follow-up time	CHD	Controls
All cancer patients		
Mean, years (SD)	**9.2±9.3***	**7.8±7.8***
Median, years (IQR)	6.3 (1.9-14.5)	5.3 (2.0-11.3)
Patients with cancer who died		
Mean, years (SD)	3.3±6.6	2.9±4.9
Median, years (IQR)	**0.8 (0.2-3.3)***	**1.3 (0.7-3.0)***
Patients with cancer who died (excl. syndromes and transplant recipients)		
Mean, years (SD)	3.7±7.1	2.8±4.6
Median, years (IQR)	1.0 (0.3-3.6)	1.2 (0.7-2.9)

**Numbers in bold denotes significance (p <0.05)*

Supplementary table 7. Cancer diagnosis in patients with CHD and controls who died (all patients)

Cancer diagnosis	Cases	Controls
Malignant neoplasms, stated or presumed to be primary, of lymphoid, haematopoietic and related tissue	51 (36.7%)	87 (20,4%)
Malignant neoplasms of eye, brain and other parts of central nervous system	23 (16.5%)	124 (29.0%)
Malignant neoplasms of digestive organs	16 (11.5%)	46 (10.8%)
Malignant neoplasms of urinary tract	<10	13 (3.0%)
Malignant neoplasms of mesothelial and soft tissue	<10	36 (8.4%)
Malignant neoplasms of ill-defined, secondary and unspecified sites	<10	15 (3.5%)
Malignant neoplasms of lip, oral cavity and pharynx	<10	11 (2.6%)
Malignant neoplasms of breast	<10	24 (5.6%)
Malignant neoplasms of female genital organs	<5	14 (3.3%)
Malignant neoplasms of bone and articular cartilage	<5	28 (6.6%)
Melanoma and other malignant neoplasms of skin	<5	20 (4.7%)
Malignant neoplasms of male genital organs	<5	<5
Malignant neoplasms of thyroid and other endocrine glands	0	<5
Malignant neoplasms of respiratory and intrathoracic organs	0	<5
Malignant neoplasms of independent (primary) multiple sites	0	0

For peer review only

Supplementary table 8. Cancer diagnosis in patients with CHD and controls who died (excluding syndromes and transplant recipients)

Cancer diagnosis	Cases	Controls
Malignant neoplasms of eye, brain and other parts of central nervous system	20 (22.5%)	121 (30.0%)
Malignant neoplasms, stated or presumed to be primary, of lymphoid, haematopoietic and related tissue	18 (20.2%)	80 (19,8%)
Malignant neoplasms of digestive organs	11 (12.4%)	45 (11.1%)
Malignant neoplasms of mesothelial and soft tissue	<10	31 (7.7%)
Malignant neoplasms of breast	<10	24 (5.9%)
Malignant neoplasms of ill-defined, secondary and unspecified sites	<10	14 (3.5%)
Malignant neoplasms of lip, oral cavity and pharynx	<10	11 (2.7%)
Malignant neoplasms of urinary tract	<5	13 (3.2%)
Malignant neoplasms of female genital organs	<5	14 (3.5%)
Malignant neoplasms of bone and articular cartilage	<5	27 (6.7%)
Melanoma and other malignant neoplasms of skin	<5	17 (4.2%)
Malignant neoplasms of male genital organs	<5	<5
Malignant neoplasms of thyroid and other endocrine glands	0	<5
Malignant neoplasms of respiratory and intrathoracic organs	0	<5
Malignant neoplasms of independent (primary) multiple sites	0	0

Supplementary table 9. Deaths

Deaths	CHD	Controls
Female/Male	69/70	213/214
Birth period (patients with cancer)		
- 1970-1979	41	183
- 1980-1989	36	89
- 1990-1999	34	82
- 2000-2009	14	60
- 2010-2017	14	13
Lesion group (patients with cancer)		
- Complex lesions	35 (29.2%)	
- Non-complex lesions	104 (16.3%)	

For peer review only

Supplementary table 10. Mortality IR divided by gender, lesion groups and birth cohorts

**Mortality IR***	**CHD (incl/excl syndromes+Tx)**	**Controls (incl/excl syndromes+Tx)**
In total cancer population	1.99 / 1.72	1.49 / 1.46
Cancer population divided by:		
- ***Gender***		
Male	2.03 / 1.82	1.56 / 1.51
Female	1.95 / 1.61	1.43 / 1.41
- ***Lesion groups***		
Complex lesions	3.70 / 2.40	
Non-complex lesions	1.72 / 1.63	
- ***Birth period***		
1970-1979	1.89 / 1.78	1.46 / 1.41
1980-1989	2.05 / 1.50	1.24 / 1.19
1990-1999	1.78 / 1.43	1.60 / 1.63
2000-2009	1.52 / 1.23	1.86 / 1.90
2010-2017	6.13 / 6.88	2.54 / 2.01

* per 100 person-years

Supplementary table 11. Mortality HR divided by gender, lesion groups and birth cohorts

**Mortality hazard ratio**	**HR (95% CI)**	
Divided by gender	Incl. syndromes and tpx	Excl. syndromes and tpx
Unadjusted model	Gender 1 **1.48 (1.13-1.93)**<br>Gender 2 **1.62 (1.23-2.13)**	Gender 1 **1.36 (1.00-1.87)**<br>Gender 2 1.33 (0.95-1.86)
Model 1 (adjusted for birth period and age at cancer diagnosis)	Gender 1 **1.36 (1.04-1.79)**<br>Gender 2 **1.36 (1.03-1.80)**	Gender 1 1.27 (0.92-1.74)<br>Gender 2 1.14 (0.81-1.60)
Model 2 (adjusted for birth period, age at cancer diagnosis and comorbidities)	Gender 1 1.32 (0.99-1.74)<br>Gender 2 1.18 (0.88-1.59)	Gender 1 1.24 (0.90-1.72)<br>Gender 2 1.09 (0.77-1.54)
Divided by lesion groups	Incl. syndromes and tpx	Excl. syndromes and tpx
Unadjusted model	Complex **2.96 (1.97-4.46)**<br>Non-complex **1.33 (1.07-1.65)**	Complex **1.99 (1.12-3.55)**<br>Non-complex 1.27 (0.99-1.64)
Model 1 (adjusted for birth period, age at cancer diagnosis and gender)	Complex **2.64 (1.72-4.07)**<br>Non-complex **1.17 (0.94-1.46)**	Complex 1.76 (0.98-3.17)<br>Non-complex 1.14 (0.89-1.47)
Model 2 (adjusted for birth period, age at cancer diagnosis, gender and comorbidities)	Complex **2.32 (1.45-3.72)**<br>Non-complex 1.11 (0.88-1.39)	Complex 1.70 (0.90-3.19)<br>Non-complex 1.11 (0.86-1.44)
Divided by birth cohorts	Incl. syndromes and tpx	Excl. syndromes and tpx
Unadjusted model	1970-1989 **1.71 (1.33-2.21)**<br>1990-2017 **1.36 (1.01-1.83)**	1970-1989 **1.49 (1.11-2.00)**<br>1990-2017 1.16 (0.80-1.68)
Model 1 (adjusted for age at cancer diagnosis and gender)	1970-1989 **1.50 (1.16-1.94)**<br>1990-2017 1.16 (0.86-1.57)	1970-1989 1.32 (0.98-1.77)<br>1990-2017 1.01 (0.70-1.47)
Model 2 (adjusted for age at cancer diagnosis, gender and comorbidities)	1970-1989 **1.37 (1.05-1.80)**<br>1990-2017 1.08 (0.80-1.48)	1970-1989 1.26 (0.93-1.71)<br>1990-2017 1.00 (0.69-1.46)

For peer review only

Supplementary table 12. Age at cancer diagnosis

**All patients**	**CHD**	**Controls**
Mean, years (SD)	**16.5±14.5***	**22.7±14.2***
Median, years (IQR)	**13.0 (2.9–30.0)***	**24.6 (8.6–35.1)***
**All patients – excl. syndromes/Tx**	**CHD**	**Controls**
Mean, years (SD)	**18.5±14.8***	**23.0±14.1***
Median, years (IQR)	**16.6 (3.9-32.2)**	**25.0 (9.1-35.2)***
**Patients who died**	**CHD**	**Controls**
Mean, years (SD)	**14.2±14.1***	**17.0±13.5***
Median, years (IQR)	**9.3 (1.7-24.0)***	**14.8 (3.8-29)***
**Patients who died – excl. syndromes/Tx**	**CHD**	**Controls**
Mean, years (SD)	16.2±15.0	17.2±13.6
Median, years (IQR)	13.5 (1.6-29.9)	15.1 (3.9-29.1)

CHD=congenital heart disease. Tx= transplant recipients. SD=standard deviation. IQR=interquartile range.
Numbers in bold denotes significance (p <0.05)

Supplementary table 13. Age at death in patients with cancer

**Age at death – all patients**	**CHD**	**Controls**
Mean, years (SD)	17.5±14.8	19.8±13.9
Median, years (IQR)	**15.1 (3.6-30.7)***	**18.5 (6.1-32.7)***
**Age at death – excl. syndromes/Tx**	**CHD**	**Controls**
Mean, years (SD)	19.8±15.5	20.0±13.9
Median, years (IQR)	19.5 (4.7-35.6)	18.7 (6.1-32.7)

CHD=congenital heart disease. Tx= transplant recipients. SD=standard deviation. IQR=interquartile range.
Numbers in bold denotes significance (p <0.05)

Supplementary figure 1. Kaplan-Meier survival

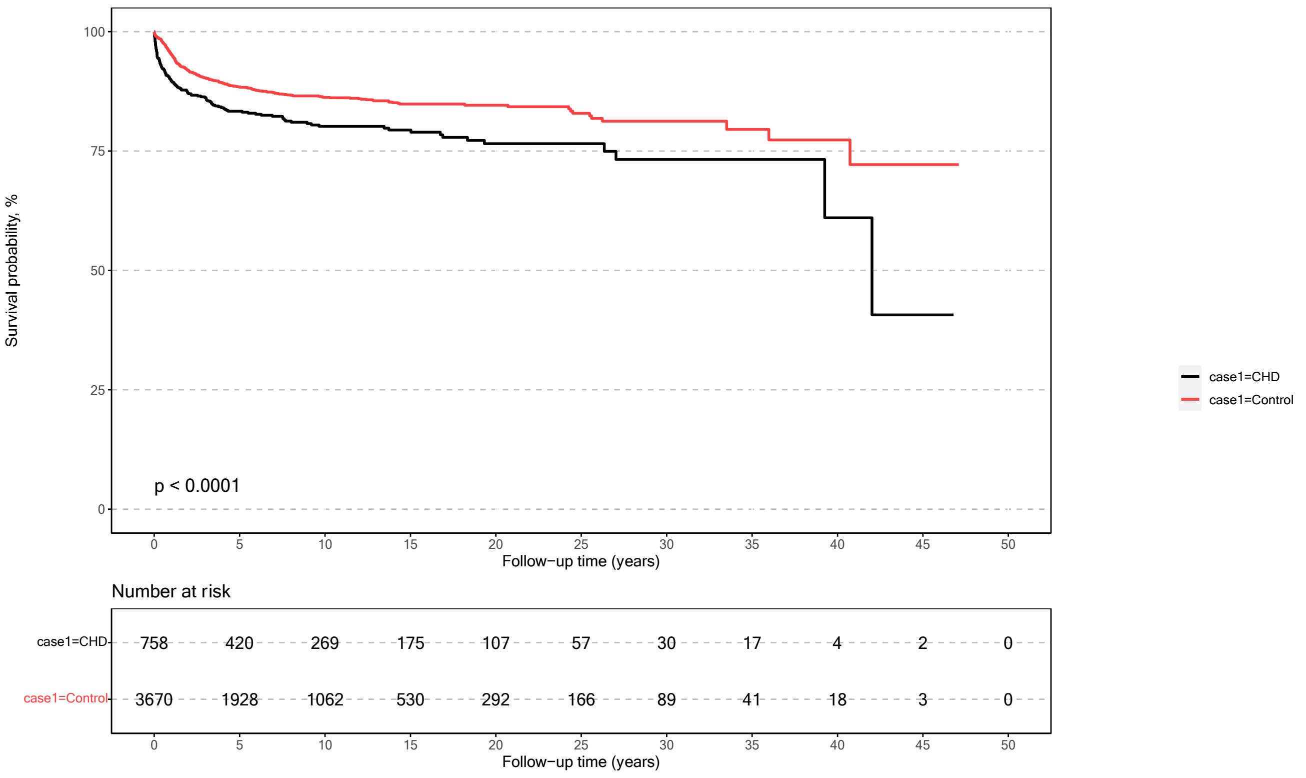

For peer revi

Supplementary figure 2a. Cause of death patients with CHD and cancer: complex lesions

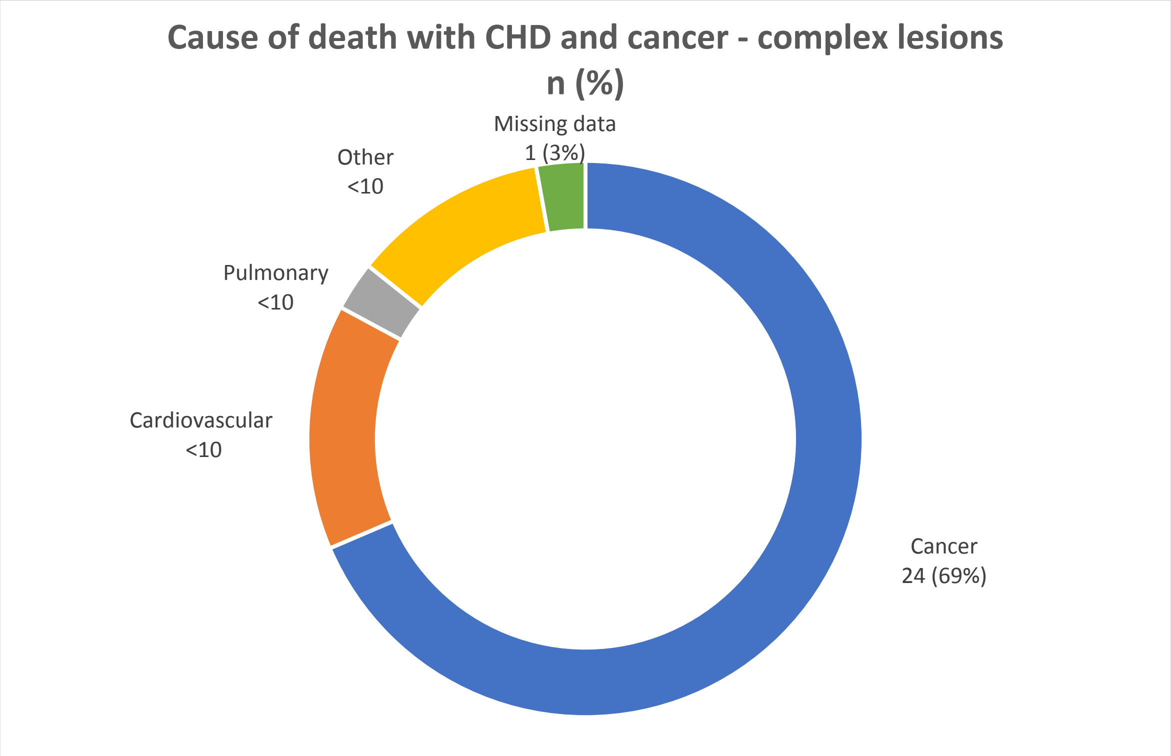

* All cause of deaths from patients and controls from the year 2017 were missing, because of later registration.

Supplementary figure 2b. Cause of death patients with CHD and cancer: non-complex lesions

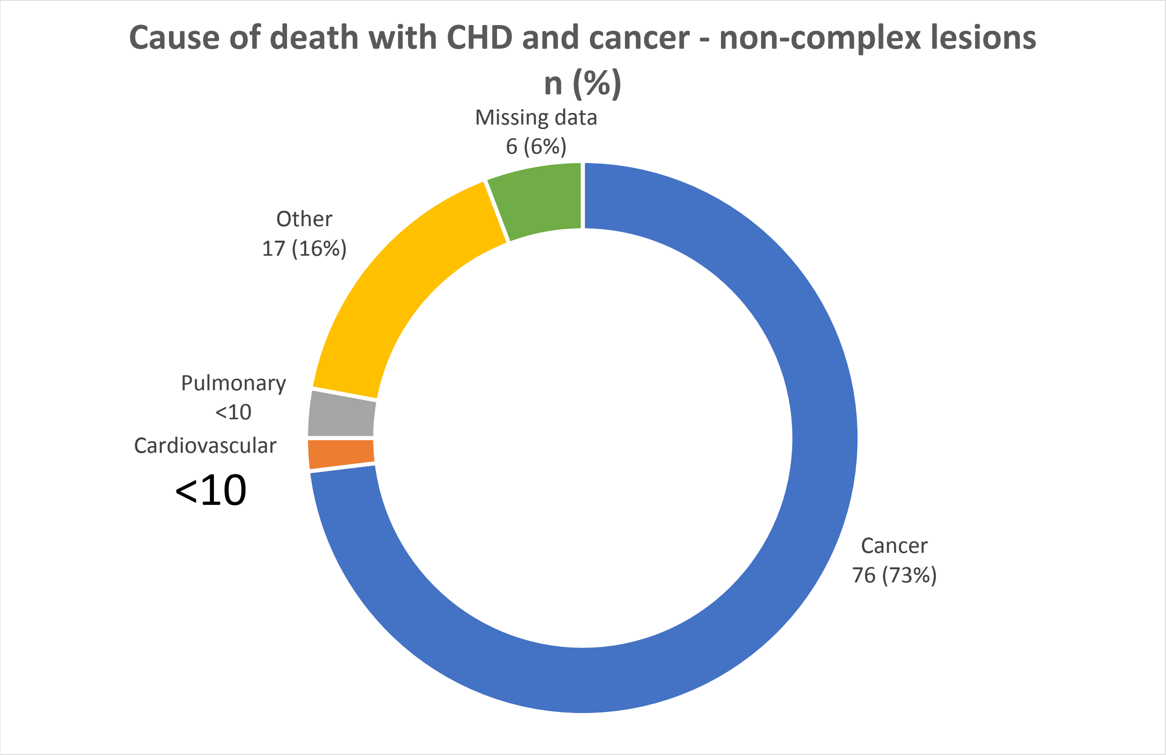

* All cause of deaths from patients and controls from the year 2017 were missing, because of later registration.

Supplementary figure 3a. Age at death in patients with CHD and cancer cause-of death (all patients)

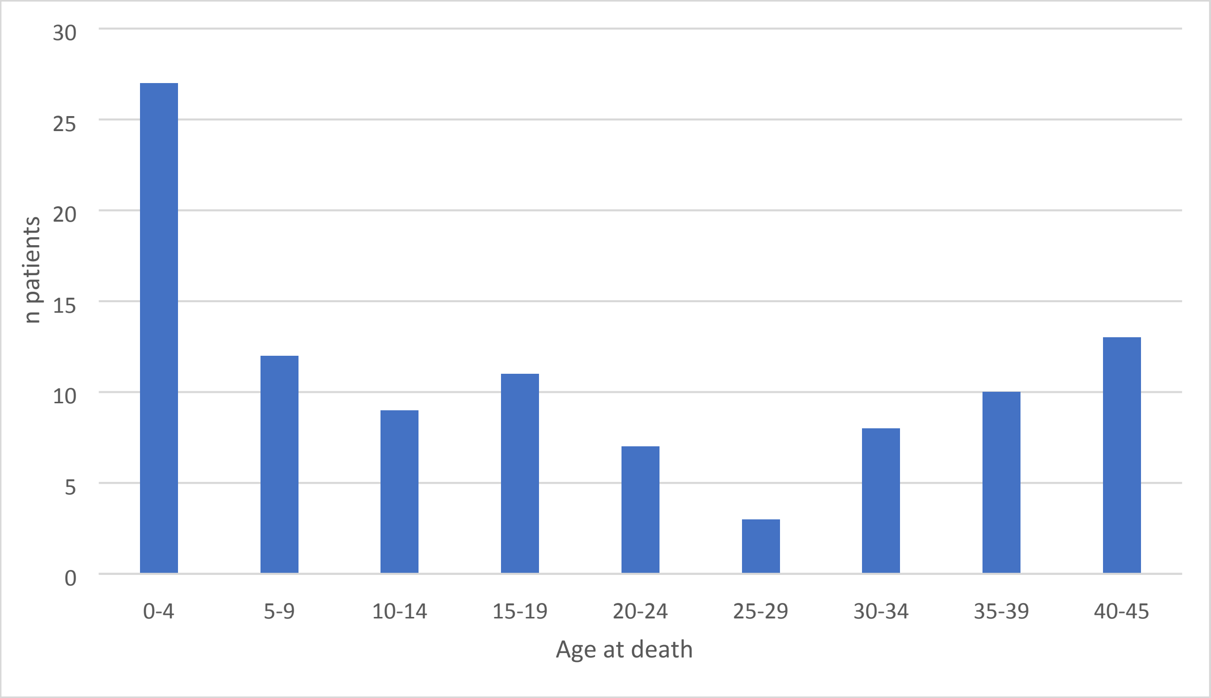

Supplementary figure 3b. Age at death in patients with CHD and cancer cause-of death (excl. syndromes and transplant recipients)

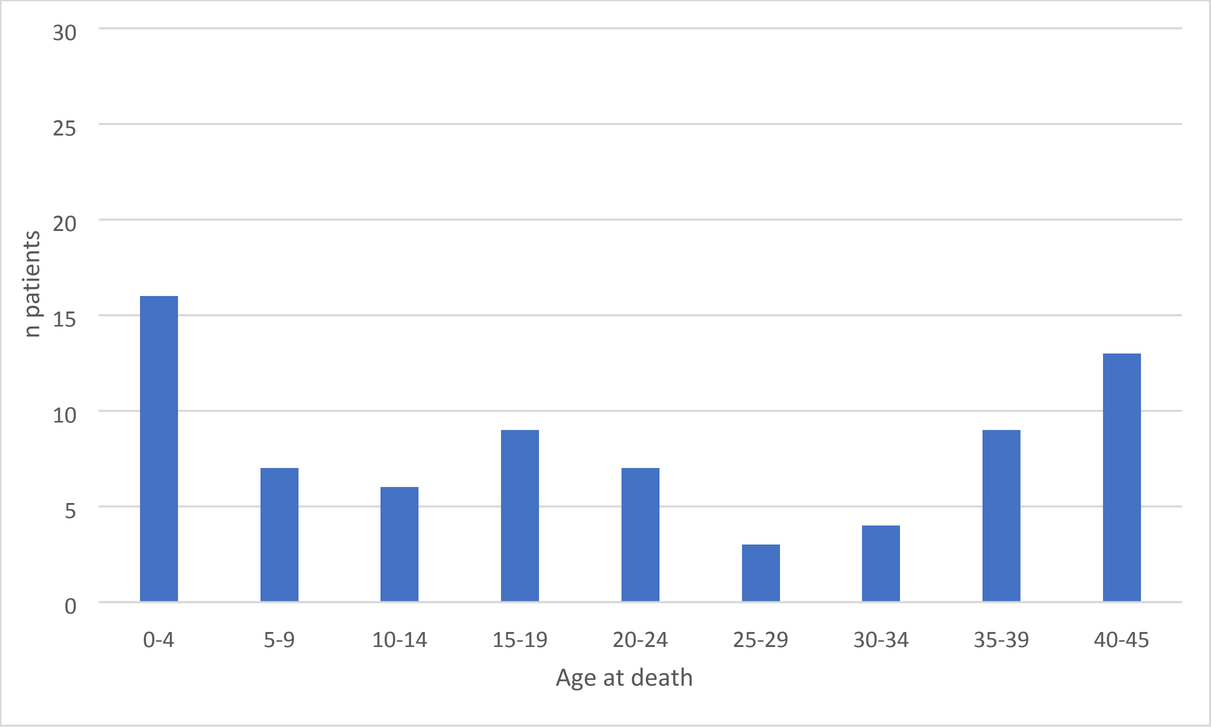

STROBE Statement—Checklist of items that should be included in reports of ***cohort studies***

	Item No	Recommendation
**Title and abstract**	1	(*a*) Indicate the study's design with a commonly used term in the title or the abstract - page 2
		(*b*) Provide in the abstract an informative and balanced summary of what was done and what was found - page 2
**Introduction**		
Background/rationale	2	Explain the scientific background and rationale for the investigation being reported - page 3
Objectives	3	State specific objectives, including any prespecified hypotheses - page 3-4
**Methods**		
Study design	4	Present key elements of study design early in the paper - page 5
Setting	5	Describe the setting, locations, and relevant dates, including periods of recruitment, exposure, follow-up, and data collection - page 5-6
Participants	6	(*a*) Give the eligibility criteria, and the sources and methods of selection of participants. Describe methods of follow-up - page 6-7
		(*b*) For matched studies, give matching criteria and number of exposed and unexposed - page 5
Variables	7	Clearly define all outcomes, exposures, predictors, potential confounders, and effect modifiers. Give diagnostic criteria, if applicable - page 6-7
Data sources/ measurement	8*	For each variable of interest, give sources of data and details of methods of assessment (measurement). Describe comparability of assessment methods if there is more than one group - page 6-7
Bias	9	Describe any efforts to address potential sources of bias - page 14
Study size	10	Explain how the study size was arrived at - page 5
Quantitative variables	11	Explain how quantitative variables were handled in the analyses. If applicable, describe which groupings were chosen and why - page 6
Statistical methods	12	(*a*) Describe all statistical methods, including those used to control for confounding - page 6-7
		(*b*) Describe any methods used to examine subgroups and interactions - page 6-7
		(*c*) Explain how missing data were addressed - page 9
		(*d*) If applicable, explain how loss to follow-up was addressed - N/A
		(*e*) Describe any sensitivity analyses - N/A
**Results**		
Participants	13*	(a) Report numbers of individuals at each stage of study—eg numbers potentially eligible, examined for eligibility, confirmed eligible, included in the study, completing follow-up, and analysed - page 7
		(b) Give reasons for non-participation at each stage - N/A
		(c) Consider use of a flow diagram -
Descriptive data	14*	(a) Give characteristics of study participants (eg demographic, clinical, social) and information on exposures and potential confounders - page 7 and table 1
		(b) Indicate number of participants with missing data for each variable of interest- page 9
		(c) Summarise follow-up time (eg, average and total amount) - page 7 and supplementary table 6
Outcome data	15*	Report numbers of outcome events or summary measures over time - page 8-10
Main results	16	(*a*) Give unadjusted estimates and, if applicable, confounder-adjusted estimates and their precision (eg, 95% confidence interval). Make clear which confounders were adjusted for and why they were included - page 8-10
		(*b*) Report category boundaries when continuous variables were categorized - page 9-10
		(*c*) If relevant, consider translating estimates of relative risk into absolute risk for a meaningful time period -

For peer review only

Other analyses	17	Report other analyses done—eg analyses of subgroups and interactions, and sensitivity analyses - page 8-10 (exclusion of patients with syndromes and transplant recipients)
**Discussion**		
Key results	18	Summarise key results with reference to study objectives - page 10-13
Limitations	19	Discuss limitations of the study, taking into account sources of potential bias or imprecision. Discuss both direction and magnitude of any potential bias - page 14
Interpretation	20	Give a cautious overall interpretation of results considering objectives, limitations, multiplicity of analyses, results from similar studies, and other relevant evidence - page 15
Generalisability	21	Discuss the generalisability (external validity) of the study results - page 14
**Other information**		
Funding	22	Give the source of funding and the role of the funders for the present study and, if applicable, for the original study on which the present article is based - page 15

*Give information separately for exposed and unexposed groups.

Note: An Explanation and Elaboration article discusses each checklist item and gives methodological background and published examples of transparent reporting. The STROBE checklist is best used in conjunction with this article (freely available on the Web sites of PLoS Medicine at http://www.plosmedicine.org/, Annals of Internal Medicine at http://www.annals.org/, and Epidemiology at http://www.epidem.com/). Information on the STROBE Initiative is available at http://www.strobe-statement.org.

For peer review only

For peer review only - http://bmjopen.bmj.com/site/about/guidelines.xhtml

BMJ Open

Outcomes after cancer diagnosis in children and adult patients with congenital heart disease in Sweden – a registry-based cohort study

Journal:	*BMJ Open*
Manuscript ID	bmjopen-2023-083237.R1
Article Type:	Original research
Date Submitted by the Author:	07-Mar-2024
Complete List of Authors:	Karazisi, Christina; University of Gothenburg, Department of Molecular and Clinical Medicine; Sahlgrenska University Hospital, Department of Medicine, Geriatrics and Emergency Medicine, Region Västra Götaland<br>Dellborg, Mikael; University of Gothenburg, Department of Molecular and Clinical Medicine; Sahlgrenska University Hospital, Department of Medicine, Geriatrics and Emergency Medicine, Region Västra Götaland<br>Mellgren, Karin; Sahlgrenska University Hospital, Department of Pediatric Oncology<br>Giang, Kok Wai; University of Gothenburg, Department of Molecular and Clinical Medicine; Sahlgrenska University Hospital, Department of Medicine, Geriatrics and Emergency Medicine, Region Västra Götaland<br>Skoglund, Kristofer; University of Gothenburg, Department of Molecular and Clinical Medicine<br>Eriksson, Peter; University of Gothenburg, Department of Molecular and Clinical Medicine; Sahlgrenska University Hospital, Department of Medicine, Geriatrics and Emergency Medicine, Region Västra Götaland<br>mandalenakis, zacharias; University of Gothenburg, Department of Molecular and Clinical Medicine; Sahlgrenska University Hospital, Department of Medicine, Geriatrics and Emergency Medicine, Region Västra Götaland
**Primary Subject Heading**:	Cardiovascular medicine
Secondary Subject Heading:	Oncology
Keywords:	Epidemiology < TROPICAL MEDICINE, Congenital heart disease < CARDIOLOGY, CARDIOLOGY, ONCOLOGY, Prognosis

SCHOLARONE™
Manuscripts

I, the Submitting Author has the right to grant and does grant on behalf of all authors of the Work (as defined in the below author licence), an exclusive licence and/or a non-exclusive licence for contributions from authors who are: i) UK Crown employees; ii) where BMJ has agreed a CC-BY licence shall apply, and/or iii) in accordance with the terms applicable for US Federal Government officers or employees acting as part of their official duties; on a worldwide, perpetual, irrevocable, royalty-free basis to BMJ Publishing Group Ltd ("BMJ") its licensees and where the relevant Journal is co-owned by BMJ to the co-owners of the Journal, to publish the Work in this journal and any other BMJ products and to exploit all rights, as set out in our licence.

The Submitting Author accepts and understands that any supply made under these terms is made by BMJ to the Submitting Author unless you are acting as an employee on behalf of your employer or a postgraduate student of an affiliated institution which is paying any applicable article publishing charge ("APC") for Open Access articles. Where the Submitting Author wishes to make the Work available on an Open Access basis (and intends to pay the relevant APC), the terms of reuse of such Open Access shall be governed by a Creative Commons licence – details of these licences and which Creative Commons licence will apply to this Work are set out in our licence referred to above.

Other than as permitted in any relevant BMJ Author's Self Archiving Policies, I confirm this Work has not been accepted for publication elsewhere, is not being considered for publication elsewhere and does not duplicate material already published. I confirm all authors consent to publication of this Work and authorise the granting of this licence.

For peer review only

Outcomes after cancer diagnosis in children and adult patients with congenital heart disease in Sweden – a registry-based cohort study

Christina Karazisi, MD,[1,2]; Mikael Dellborg, MD,[1,2,3]; Karin Mellgren, MD,[4];
Kok Wai Giang, PhD,[1,2]; Kristofer Skoglund, MD,[1]; Peter Eriksson, MD,[1,2,3];
Zacharias Mandalenakis, MD[1,2,3]

[1] Institute of Medicine, Department of Molecular and Clinical Medicine, Sahlgrenska Academy, University of Gothenburg, Sweden
[2] Department of Medicine, Geriatrics and Emergency Medicine, Region Västra Götaland, Sahlgrenska University Hospital/Östra, Gothenburg, Sweden
[3] Adult Congenital Heart Disease Unit, Sahlgrenska University Hospital, Gothenburg, Sweden
[4] Department of Pediatric Oncology, the Queen Silvia Children's Hospital, Sahlgrenska University Hospital, Gothenburg, Sweden

Corresponding author:
Christina Karazisi, MD
Department of Molecular and Clinical Medicine
Sahlgrenska University Hospital
Diagnosvägen 11, SE-416 50 Gothenburg, Sweden
E-mail: christina.karazisi@vgregion.se
Tel: +4631 342000; Fax: +4631 191416

Manuscript word count: 3756 (not including title, abstract, references)

Keywords: congenital heart disease; cardio-oncology; cancer mortality; prognosis

For peer review only - http://bmjopen.bmj.com/site/about/guidelines.xhtml

Abstract

Objective: Patients with congenital heart disease (CHD) have an increased cancer risk. The aim of this study was to determine cancer-related mortality in CHD patients compared with non-CHD controls, compare ages at cancer diagnosis and death, and explore the most fatal cancer diagnoses.

Design: Registry-based cohort study

Setting and participants: CHD patients born between 1970 and 2017 were identified using Swedish Health Registers. Each was matched by birth year and sex with 10 non-CHD controls. Included were those born in Sweden with a cancer diagnosis.

Results: Cancer developed in 758 out of 67814 CHD patients (1.1%), with 139 deaths (18.3%) – of which 41 deaths occurred in patients with syndromes. Cancer was the cause of death in 71.9% of cases. Across all CHD patients, cancer accounted for 1.8% of deaths. Excluding patients with syndromes and transplant recipients, mortality risk between CHD patients with cancer and controls showed no significant difference (HR 1.17; 95% CI 0.93–1.49). CHD patients had a lower median age at cancer diagnosis—13.0 years (IQR 2.9–30.0) in CHD vs. 24.6 years (IQR 8.6–35.1) in controls. Median age at death was 15.1 years (IQR 3.6–30.7) in CHD patients vs. 18.5 years (IQR 6.1–32.7) in controls. The top three fatal cancer diagnoses were ill-defined, secondary and unspecified, CNS tumors and hematologic malignancies.

Conclusions: Cancer-related deaths constituted 1.8% of all mortalities across all CHD patients. Among CHD patients with cancer, 18.3% died, with cancer being the cause in 71.9% of cases. Although CHD patients have an increased cancer risk, their mortality risk post-

For peer review only - http://bmjopen.bmj.com/site/about/guidelines.xhtml

diagnosis does not significantly differ from non-CHD patients when excluding patients with syndromes and transplant recipients. However, CHD patients with syndromes and concurrent cancer appear to be a vulnerable group.

Strengths and limitations of this study

- The primary strength of this study lies in its nationwide scope; it includes all patients born in Sweden between 1970 and 2017 who has been diagnosed with CHD.
- The follow-up is from birth, in a country with universal health coverage.
- A major limitation is that it relies solely on register-based data, which excludes clinical details. Consequently, we lacked specific information regarding the cancer treatment administered to patients.
- Moreover, we did not have information on cancer stages.

For peer review only

Introduction

Nearly 2% of children are born with a congenital heart defect (1). These defects range from simple and uncomplicated, sometimes resolving with age, to extremely complex conditions necessitating multiple surgeries and/or catheter interventions for survival. The prognosis for congenital heart disease (CHD) has improved significantly, with over 97% now expected to reach adulthood (2).

In previous studies, we have found that the overall cancer risk for CHD patients in Sweden is higher than in matched controls without CHD. Notably, this risk in children and young adults is more than double that of the matched controls (3, 4). Some syndromes are known to have an increased risk of both CHD and cancer, such as Downs and 22q11-deletion syndrome (5, 6). Patients with Fontan circulation have been shown over time to develop hepatic fibrosis or cirrhosis, with risk of hepatocellular carcinoma (7, 8). An association has also been found between cyanotic CHD and an increased risk of pheochromocytomas and paragangliomas (9, 10).

Several studies have tried to find potential risk factors for cancer in CHD patients. Thymectomy at young age can be a possible risk factor (4, 11). Heart transplantation is possible for CHD patients with end-stage circulatory failure (12). Studies have shown that transplant recipients have a more than two-fold overall increase of cancer compared to the general population (13, 14). The association between low-dose ionizing radiation and cancer risk in CHD patients have also been studied. However, in studies with calculation of radiation doses and adjustment or exclusion of cancer predisposition syndromes and/or transplantation – no association has been found (15, 16). Most likely, the association between CHD and cancer is multifactorial. As age increases, so does the risk of acquired diseases such as cancer.

For peer review only

Furthermore, evidence suggests that CHD patients are less likely to participate in screenings for malignancies, including cervical, breast, and colon cancer (17). This raises the question if cancer is found at a later stage in CHD patients, which can affect survival rate. Additionally, given that CHD patients have a higher risk of heart failure compared to controls (18), this could possibly affect cancer treatment - as this can theoretically put the patient at risk of cardiotoxicity – which also could affect the risk of mortality.

Cancer ranks as the fourth leading cause of death among adult CHD patients, preceded by heart failure, pneumonia, and sudden cardiac death (19). Venkatesh et al. recently reported on malignancy outcomes in a single-center cohort comprising 68 adults with CHD and cancer. In this group, 24% of the patients died during a median follow-up period of five years, with cancer being the cause of death in 69% of these cases (20). However, to our knowledge, there has been no studies exploring whether there is an elevated risk of mortality in CHD patients with cancer compared with cancer patients without CHD. Furthermore, there are no studies on long-term outcomes for children with CHD following a cancer diagnosis.

In this study, we aim to investigate the prevalence of cancer-related mortality in pediatric and adult patients with CHD born in Sweden between 1970 and 2017. We also examine whether there is an increased risk of mortality in patients with CHD who also have cancer, compared with a control group of cancer patients without CHD. In addition, we will investigate whether the age at cancer diagnosis and mortality differs between patients with CHD and those without CHD. Furthermore, we will identify the most common fatal cancer diagnoses in this population.

For peer review only

Methods

Study population and design

This nationwide registry-based cohort study utilized data from the Swedish National Inpatient Register (established in 1964, encompassing all cardiothoracic centers since 1970, and achieving national coverage in 1987), the Swedish National Outpatient Register (complete since 2001) and the Swedish Cause of Death Register (complete since 1968). The International Statistical Classification of Diseases and Related Health Problems (ICD) facilitated the identification of diagnoses. Controls were sourced from the Swedish Total Population Register, which is a register where all Swedish inhabitants are registered in since 1968.

Patients diagnosed with CHD born between 1970 and 2017 were identified for the study. Each CHD patient was paired with 10 controls *without* CHD, matched by sex and birth year. Patients not born in Sweden were excluded. The study included 67814 and 583709 controls; among these, we identified individuals diagnosed with cancer (758 CHD patients and 3670 controls) for inclusion. The Swedish National Board of Health and Welfare and Statistics Sweden provided case-control identification and facilitated register linkage. In the final dataset, all national registration numbers were replaced with a code, ensuring data anonymization. Data analysis was conducted from January 2023 to October 2023.

Ethics approval

This study was approved by the Gothenburg Regional Research Ethics Board (Gbg 912-16, T 619-18). The tenets of the Declaration of Helsinki were followed, and the need for patient consent was waived as the study utilized anonymized register-based data. This report follows

For peer review only - http://bmjopen.bmj.com/site/about/guidelines.xhtml

the Strengthening the Reporting of Observational Studies in Epidemiology (STROBE) reporting guidelines.

Definitions

CHD was defined using ICD codes, as outlined in Supplementary Table 1. The diagnoses of CHD were classified in accordance with the hierarchical CHD classification system initially described by Botto et al. and later modified by Liu et al (21, 22). Patients were divided into two groups: those with complex lesions (lesion group 1–2) and those with non-complex lesions (lesion group 3–6) (Supplementary Table 2). Cancer was defined using ICD codes, as specified in Supplementary Table 3. Syndromes were defined using ICD codes, detailed in Supplementary Table 4. Comorbidities were defined using ICD codes according to Supplementary Table 5, prior to the date of cancer diagnosis. Transplant recipients in this article refers to transplant recipients of heart, heart/lung, kidney and liver.

Statistical analysis

All statistical analyses were performed using R v3.5.2 (R Foundation for Statistical Computing, Vienna, Austria). Baseline characteristics of patients are described with categorical data shown as numbers and percentages, while continuous data are presented as mean ± standard deviation (SD) or median and interquartile range (IQR). Descriptive statistics were used to report cancer-related mortality among all patients with CHD, expressed as numbers and percentages. The patients were observed from cancer diagnosis until death or the study's conclusion (December 31, 2017).

Mortality incidence rates (IR) were reported per 100 person-years, calculated as the total deaths divided by the cumulative follow-up time of the study population. Hazard ratios (HR)

For peer review only - http://bmjopen.bmj.com/site/about/guidelines.xhtml

with 95% CIs were derived using Cox proportional hazard regression models. In these models, the control group served as the reference. The analysis was initially conducted without adjustments, followed by two adjusted models. The first adjusted model accounted for variables such as birth year, age at cancer diagnosis, and gender. The second adjusted model included the variables from the first model and additionally adjusted for comorbidities, namely hypertension, diabetes, myocardial infarction, ischemic stroke, atrial fibrillation, and heart failure. A p-value of less than 0.05 was considered statistically significant.

Patient and public involvement

None.

Results

Baseline characteristics

We identified 67814 patients born in Sweden between 1970 and 2017 who had been diagnosed with CHD, along with 583,709 matched controls without CHD who were matched by birth year and sex. Among these, 758 (1.1%) CHD patients and 3670 (0.6%) controls subsequently developed cancer. The characteristics of our study population are shown in Table 1. Cancer cases were most commonly found in the eldest birth cohort 1970-1979 in both CHD patients and controls. However, in the CHD patients the proportion of patients in this group was smaller than in the control group (29.3% vs 41.9%) and more evenly distributed between the birth cohorts. The proportion of CHD patients that was diagnosed with first cancer diagnosis at an age below 18 years was larger than in controls (59.5% vs 38.6%). As expected, patients with syndromes were more commonly found in CHD patients than in controls (21% vs 2.0%). Most comorbidities were also more frequently found in CHD patients. The median follow-up duration post-cancer diagnosis was 6.3 years (IQR 1.9–14.5)

For peer review only - http://bmjopen.bmj.com/site/about/guidelines.xhtml
For peer review only

for CHD patients and 5.3 years (IQR 2.0–11.3) for the control group (Supplementary Table 6). The median age at last follow-up was 25.7 years (IQR 13.8-38.5) for CHD patients and 33.5 years (20.5-42.5) for the control group.

Table 1. Study population

Characteristic	CHD	Controls
All patients*, *No.	67814	583709
Patients with cancer, No. (% of total)	758 (1.1%)	3670 (0.6%)
- Male	360 (47.5%)	1663 (45.3%)
- Female	398 (52.5%)	2007 (54.7%)
Birth period (patients with cancer), No. (%)		
- 1970–1979	222 (29.3%)	1538 (41.9%)
- 1980–1989	158 (20.8%)	832 (22.7%)
- 1990–1999	169 (22.3%)	616 (16.8%)
- 2000–2009	130 (17.2%)	489 (13.3%)
- 2010–2017	79 (10.4%)	195 (5.3%)
Age at cancer diagnosis, No (%)		
- 0-17 years	451 (59.5%)	1416 (38.6%)
- 18+	307 (40.5%)	2254 (61.4%)
Lesion group (patients with cancer), No. (%)		
- Complex lesions	120 (15.8%)	
- Non-complex lesions	638 (84.2%)	
Comorbidities (patients with cancer), No. (%)		
- Hypertension	27 (3.6%)	44 (1.2%)
- Diabetes	12 (1.6%)	42 (1.1%)
- Myocardial infarction	1 (0.1%)	3 (0.1%)
- Ischemic stroke	8 (1.1%)	7 (0.2%)
- Atrial fibrillation	21 (2.8%)	10 (0.3%)
- Heart failure	46 (6.1%)	6 (0.2%)
Syndromes, n (%)	159 (21%)	75 (2.0%)
- **Complex**	42	61
- **Non-complex**	117	14
Transplant recipients, n (%)	36 (4.7%)	23 (0.6%)

For peer review only

Cancer diagnoses

Figure 1a–b shows the distribution of cancer diagnoses among CHD patients and controls. Among patients who died, the three predominant types diagnosed in those with CHD were hematologic (28.5%), ill-defined, secondary and unspecified sites (20.8%) and CNS tumors (12.6%). In controls, ill-defined, secondary and unspecified sites were the most common (26.0%), followed by CNS tumors (19.3%) and hematologic malignancies (15.4%) (Supplementary Table 7). Upon excluding individuals with syndromes and those who had undergone transplants, ill-defined, secondary and unspecified sites emerged as the leading cancer diagnosis in CHD patients, followed by CNS tumors and hematologic cancer, a trend consistent in both groups (Supplementary Table 8). When calculating case fatality-rates of the different cancer types, the only cancer type that differed significantly between CHD patients and the controls was hematologic cancers when syndromes and transplant recipients were included – with 21.5% fatality rate in CHD patients vs. 14.6% in controls. When including patients with syndromes and transplant recipients the difference was no longer significant (13.2% in CHD patients vs 14.0% in controls).

Mortality after cancer diagnosis

Among the patients studied, 139 patients (18.3%) with concurrent CHD and cancer died during the study period compared with 427 (11.6%) of the control group, who had cancer but not CHD (refer to Table 2 and Supplementary Table 9 for additional details). Kaplan–Meier survival analysis for patients with cancer, categorized by the presence or absence of CHD, is presented in Supplementary Figure 1. Among the 139 deceased patients, 35 (25.2%) had complex lesions, whereas 104 (74.8%) had non-complex lesions. Consequently, the mortality rate was 29.2% among patients with complex lesions and cancer, compared with 16.3% for patients with cancer having non-complex lesions.

For peer review only - http://bmjopen.bmj.com/site/about/guidelines.xhtml
For peer review only

Table 2. Deaths

	CHD		Controls	
**Patients with syndromes/transplant recipients -->**	**Included**	**Excluded**	**Included**	**Excluded**
**Total deaths:**				
In total population, No. (%)	5689 (0.08%)	4498 (0.08%)	4117 (0.007%)	4000 (0.007%)
In cancer patients (complex/non-complex), No.	139 (35/104)	89 (14/75)	427	404
% of deaths in cancer patients	18,3%	15,8%	11.6%	11.3%
In patients with syndromes (complex/non-complex), No	41 (15/26)		17 (-)	
% of deaths in cancer patients	5,4%		0,5%	
Transplant recipients	<10		<10	
% of deaths in cancer patients	<2%		<1%	
**Cancer cause of death***				
Total deaths in which cause of death was cancer, No.	100	68	370	355
Complex lesions, No (%)	24 (24%)	8 (11,8%)	-	-
Non-complex lesions, No (%)	76 (76%)	60 (88,2%)	-	-
In patients with syndromes, No (%) (complex/non-complex)	26 (26%) (11/15)		15 (0,4%) (-)	
Transplant recipients	<10		<5	
Percentage of cancer-related death				
In total population deaths	1,8%	1,5%	9,0%	8,9%
In cancer patients	13,3%	12,1%	10,1%	9,9%
In cancer patients deaths	71,9%	76.4%	86,7%	87,9%

*Excluding patients with missing causes of death.

Patients with CHD and cancer exhibited a higher mortality IR compared with controls diagnosed with cancer but without CHD (Supplementary Table 10). Notably, the most pronounced differences in mortality IRs were observed in the youngest birth cohort, spanning 2010–2017, in which the IR for CHD patients was 6.13, in contrast to 2.54 for controls.

The initial unadjusted analysis showed a higher mortality risk following cancer diagnosis in patients with CHD compared with controls without CHD (HR 1.55; 95% CI 1.28–1.88). This

For peer review only - http://bmjopen.bmj.com/site/about/guidelines.xhtml

increased risk persisted, albeit to a lesser extent, after excluding patients with syndromes and transplant recipients (HR 1.35; 95% CI 1.08–1.7) (Table 3). Subsequent analyses, adjusting for birth period, age at cancer diagnosis, and gender in the first model, and additionally for comorbidities in the second model, revealed that the increased risk remained significant (model 1: HR 1.38; 95% CI 1.13–1.68; model 2: HR 1.27; 95% CI 1.04–1.55). However, upon the exclusion of patients with syndromes and transplant recipients, the difference in mortality risk between patients with CHD and the control group was not statistically significant (model 1: HR 1.21; 95% CI 0.96–1.53; model 2: HR 1.17; 95% CI 0.93–1.49) (Table 3, Supplementary Table 11).

Table 3. Mortality after cancer diagnosis

**Mortality hazard ratio**	**HR (95% CI)**	
All patients	Incl. syndromes and tpx	Excl. syndromes and tpx
Unadjusted model	**1.55 (1.28–1.88)***	**1.35 (1.08–1.70)***
Model 1 (adjusted for birth period, age at cancer diagnosis, and gender)	**1.38 (1.13–1.67)***	1.21 (0.96–1.53)
Model 2 (adjusted for birth period, age at cancer diagnosis, gender, and comorbidities)	**1.27 (1.03–1.55)***	1.17 (0.93–1.49)

****Numbers in bold denotes significance (p < 0.05).***
Abbreviation: tpx, transplant recipients

Cause of death

Cancer accounted for 1.8% of mortality cases in the overall CHD cohort, while it represented 72% of deaths among patients with both CHD and cancer (Table 2). In fewer than 10 instances among CHD patients with cancer, the cause of death was cardiovascular. Causes of death categorized by complex lesions and non-complex lesions are presented in

For peer review only - http://bmjopen.bmj.com/site/about/guidelines.xhtml

Supplementary Figure 2a–b. Among patients with cancer as the cause of death, 26% (n = 26) also had a syndrome diagnosis. All cause of deaths from patients and controls from the year 2017 were missing, because of later registration. Therefore, seven patients who died 2017 had missing data of cause of death.

Age at cancer diagnosis

The analysis revealed a notable difference in the age at which cancer was diagnosed between patients with CHD and the control group. Specifically, the median age for cancer diagnosis in patients with CHD was 13.0 years (IQR 2.9–30.0), whereas it was 24.6 years (IQR 8.6–35.1) in the control group (Supplementary table 12). This difference persisted even after the exclusion of patients with syndromes and transplant recipients. However, among patients with cancer who died, the initial observed difference in the age at cancer diagnosis between CHD patients and controls was no longer evident after the exclusion of patients with syndromes and transplant recipients (Supplementary Table 12).

Age at death

The median age at death was lower in patients with CHD and cancer—15.1 years (IQR 3.6–30.7) compared with 18.5 years (IQR 6.1–32.7) in controls with cancer. Excluding patients with syndromes and transplant recipients, the difference in age at death between CHD patients and controls was minimal, with a median age of 19.5 years (IQR 4.7–35.6) for CHD patients versus 18.7 years (IQR 6.1–32.7) for controls (Supplementary Table 13). Supplementary Figure 3a–b illustrates the distribution of age at death among patients with CHD and cancer.

For peer review only - http://bmjopen.bmj.com/site/about/guidelines.xhtml

Discussion

This study, to the best of our knowledge, represents the first to explore long-term outcomes following a cancer diagnosis in individuals with CHD, ranging in age from birth to a maximum of 47 years. Our analysis revealed that cancer developed in 758 (1.1%) of the patients with CHD and in 3670 (0.6%) of the controls. In CHD patients, the proportion of patients born in the eldest birth cohort was smaller than in controls: 29.3% of cancer patients in CHD patients vs 41.9% of cancer patients in controls. This probably reflects the increased survival in CHD patients; they now more frequently live long enough to develop cancer. This might also have influenced that the proportion of CHD patients diagnosed with cancer below 18 years of age was higher than those above 18 years.

In previous studies, we demonstrated that the most common cancer types in CHD patients were largely similar to those in the control group, with the exclusion of patients with syndromes and transplant recipients (4). In this study, the most frequent cancer diagnoses for both CHD patients and controls were hematologic, ill-defined, secondary and unspecified sites, skin and CNS tumors. The three leading causes of cancer-related fatalities were ill-defined, secondary and unspecified sites, followed by CNS tumors and hematologic cancer.

In a nationwide cohort study conducted in Taiwan, Lee et al. identified an elevated risk of various cancers, particularly hematologic, CNS, and head and neck malignancies, in children and adults with CHD (23). Venkatesh et al., focusing on adults with CHD who developed cancer, reported the most frequent cancer types as hematologic, breast, skin, hepatocellular, and urologic cancers (20). Notably, in that study, five out of the seven patients with hepatocellular carcinoma (HCC) had previously undergone Fontan palliation, a procedure associated with an increased HCC risk (24). In our study, we categorized HCC under

For peer review only - http://bmjopen.bmj.com/site/about/guidelines.xhtml

digestive organ malignancies, which emerged as one of the fourth most fatal cancers. However, fewer than five CHD patients who succumbed to their illnesses had HCC. This lower incidence in our study might be attributed to the younger age of our patients, considering that HCC risk notably increases over 30 years post-Fontan operation (24).

During the study, 139 patients with CHD and cancer died, constituting 18.3% of the study population. Among these, cancer was the primary cause of death for 72% of patients with CHD. This finding aligns with the research by Venkatesh et al. on adult CHD patients, which reported a 24% mortality rate among cancer patients over a median follow-up period of 5 years post-cancer diagnosis, with cancer accounting for 69% of these deaths (20).

In our total CHD cohort, 1.8% of all deaths were attributed to cancer. Previous studies have reported higher frequencies of cancer-related mortality in CHD patients, ranging from 6%–16% (19, 25, 26). However, these studies focused exclusively on adult CHD patients. As age increases, the risk of acquiring other diseases also rises, potentially affecting mortality risk. For instance, in the study reporting 16% cancer-caused mortality, 42% of the patients had heart failure (26). Conversely, in our CHD and cancer cohort, only 6.1% of patients had heart failure.

A recent study investigating causes of death in infants, children, and young adults up to 20 years old with CHD found that the primary cause of mortality in infants was CHD, followed by lung disease and infection. In children, the leading causes were CHD, neurologic disease, and infection (27). The absence of cancer in this report likely reflects its relatively low incidence of cancer-related death in pediatric patients compared with adults. Our study, encompassing both children and adult patients, suggests that the generally lower frequency of

For peer review only - http://bmjopen.bmj.com/site/about/guidelines.xhtml

For peer review only

cancer-related deaths in children might contribute to the reduced frequency of cancer-caused mortality observed in our cohort.

The mortality IRs were notably higher in CHD patients with cancer than in controls with cancer but without CHD. Overall, patients with CHD have an increased mortality risk relative to controls (28). The highest mortality IR was found in the youngest birth cohort of patients with cancer. However, this is probably due to the low numbers and the fact that this group was the smallest in comparison to the other birth cohorts and generates the smallest cumulative follow-up time as it also is the youngest.

In our analysis of whether patients *with* CHD *and* cancer face a heightened mortality risk compared with controls *without CHD* but *with* cancer, we found an increased risk in patients with CHD when factors such as birth period, age at cancer diagnosis, gender, and comorbidities were accounted for (HR 1.27; 95% CI 1.04–1.55). However, this increased risk was not significant upon excluding patients with syndromes and transplant recipients from the analysis.

Mortality rates among patients with CHD have declined over time, especially for those born after 1975 (28). Our study included patients born after 1970 who were diagnosed with cancer. Increased survival rates into adulthood may have altered both the risk of cancer development and the effectiveness of cancer treatment over time. This could explain the lack of a significant difference in survival rates between CHD patients and controls.

In the observed cohort, syndromes accounted for 41 deaths of the total 139 fatalities (29%), of which 26 were attributed to cancer (representing 26% of all cancer-related deaths among

For peer review only - http://bmjopen.bmj.com/site/about/guidelines.xhtml

CHD patients). Among these 26 cancer-related deaths, 11 occurred in patients with complex lesions (accounting for 46% of cancer-related deaths in this subgroup) and 15 in those with non-complex lesions (constituting 20% of the deaths in this category). Previous studies have shown that children and adolescents with non-Hodgkin lymphoma with pre-existing conditions such as cancer predisposition syndromes (e.g. Down syndrome), immunodeficiencies, genetic disorders (e.g. Williams-Beuren syndrome), and other non-classifiable conditions, exhibit lower survival rates compared with their counterparts without such pre-existing conditions (29). Our findings corroborate the heightened vulnerability of patients with syndromes and concurrent cancer. Case fatality rate of hematologic cancer was increased in CHD patients compared to controls when including syndromes and transplant recipients.

In examining the age at cancer diagnosis, it was observed that patients with CHD were diagnosed at a generally younger age than those without CHD. Differences were noted in the age at diagnosis among patients that died, excluding those with syndromes and transplant recipients. Recognizing the heightened risk of cancer at younger ages is crucial for timely detection and treatment, ultimately enhancing survival outcomes.

Strengths and limitations

The primary strength of our study lies in its nationwide scope; it includes all patients born in Sweden between 1970 and 2017 who had been diagnosed with CHD. Additionally, the longitudinal tracking of patients from birth enhances the robustness of our findings.

However, our study is not without limitations. It relies solely on register-based data. The Swedish Registers were not complete until 1987. Hence, that there may be missing data for

For peer review only

For peer review only - http://bmjopen.bmj.com/site/about/guidelines.xhtml

some patients and controls until the time of full coverage. Furthermore, the Registers excludes clinical details. Consequently, we lacked specific information regarding the cancer treatment administered to patients. It is important to note that cancer treatment has evolved over time. Moreover, we did not have access to information on cancer stages other than the diagnosis of ill-defined, secondary and unspecified sites – which not surprisingly, emerged as the most frequent cancer diagnosis in patients who succumbed to their illness.

Another limitation is our focus on cancer mortality rates without considering the diverse types of cancer present in the study group. The heterogeneity in cancer types and their therapies significantly influence survival and mortality rates. Despite these variations, the cancer types in CHD patients and controls exhibited similar frequency patterns, lending a degree of reliability to our findings. Furthermore, since we do not have access to any results of genetic testing, the proportion of genetic disorders might be underestimated. Additionally, it is important to note that Sweden has a universal health coverage system. Consequently, these results may not be generalizable to patients with CHD in countries with different health care systems.

Conclusions

In conclusion, this study revealed that only 1.8% of deaths among Swedish patients with CHD, with or without cancer, were attributable to cancer. Among those with both CHD and cancer, 18.3% died within a median follow-up duration of 6.3 years (IQR 1.9–14.5). In the CHD patients who died, cancer was identified as the cause of death in 71.9% of cases. Notably, the mortality risk in CHD patients with cancer did not significantly exceed that of controls with cancer alone, once patients with syndromes and transplant recipients were excluded from the analysis. These findings indicate that while CHD patients face an increased

For peer review only

risk of developing cancer and tend to develop cancer at a younger age, their mortality risk post-cancer diagnosis does not significantly differ from that of patients without CHD, again excluding those with syndromes and transplant recipients.

Acknowledgment: We thank Phoebe Chi, MD, from Edanz (https://www.edanz.com/ac), for editing a draft of this manuscript.

Author contribution: All authors have contributed significantly to the submitted work. CK, MD, KM, KWG, KS, PE and ZM contributed to the conception and design of the study. CK, ZM and KWG did the experimental design and the analyses. CK and ZM prepared the manuscript and verified the data. CK, MD, KM, KWG, KS, PE and ZM critically reviewed the manuscript and approved the final manuscript. All authors had full access to all the data in the study and had final responsibility for the decision to submit for publication.

Funding/Support: The study was funded by the Swedish state under an agreement between the Swedish Government and County Councils (Grant Number: ALFGBG-955256), the Swedish Research Council (Grant Number: 2019-00193), the Swedish Heart-Lung Foundation (Grant Number: 20190724), and the Swedish Childhood Cancer Fund (Grant Number: SP2017-0012).

Conflict of Interest Disclosures: None reported.

Data availability statement: Permission to access the data must be applied for through a research ethics board and from the primary data owners, the Swedish National Board of Health and Welfare and Statistics Sweden.

For peer review only

References

1. Giang KW, Mandalenakis Z, Fedchenko M, et al. Congenital heart disease: changes in recorded birth prevalence and cardiac interventions over the past half-century in Sweden. *Eur J Prev Cardiol*. 2023;30(2):169-76.

2. Mandalenakis Z, Giang KW, Eriksson P, et al. Survival in children with congenital heart disease: Have we reached a peak at 97%? *J Am Heart Assoc*. 2020;9(22):e017704.

3. Mandalenakis Z, Karazisi C, Skoglund K, et al. Risk of cancer among children and young adults with congenital heart disease compared with healthy controls. *JAMA Netw Open.* 2019;2(7):e196762.

4. Karazisi C, Dellborg M, Mellgren K, et al. Risk of cancer in young and older patients with congenital heart disease and the excess risk of cancer by syndromes, organ transplantation and cardiac surgery: Swedish health registry study (1930-2017). *Lancet Reg Health Eur*. 2022;18:100407.

5. Patja K, Pukkala E, Sund R, et al. Cancer incidence of persons with Down syndrome in Finland: a population-based study. Int J Cancer. 2006;118(7):1769-72.

6. Stevens T, van der Werff Ten Bosch J, De Rademaeker M, et al. Risk of malignancy in 22q11.2 deletion syndrome. Clin Case Rep. 2017;5(4):486-90.

7. Emamaullee J, Zaidi AN, Schiano T et al. Fontan-Associated Liver Disease: Screening, Management, and Transplant Considerations. Circulation. 2020;142(6):591-604.

8. Possner M, Gordon-Walker T, Egbe AC et al. Hepatocellular carcinoma and the Fontan circulation: Clinical presentation and outcomes. Int J Cardiol. 2021;322:142-8.

9. Opotowsky AR, Moko LE, Ginns J et al. Pheochromocytoma and paraganglioma in cyanotic congenital heart disease. J Clin Endocrinol Metab. 2015;100(4):1325-34.

10. Adunuri N, Mrkobrada M. Not So Incidental: Pheochromocytoma in an Adult with Unrepaired Cyanotic Heart Disease. Eur J Case Rep Intern Med. 2018;5(10):000959.

11. Gudmundsdottir J, Söderling J, Berggren H et al. Long-term clinical effects of early thymectomy: Associations with autoimmune diseases, cancer, infections, and atopic diseases. J Allergy Clin Immunol. 2018;141(6):2294-7.e8.
12. Ross HJ, Law Y, Book WM et al. Transplantation and Mechanical Circulatory Support in Congenital Heart Disease: A Scientific Statement From the American Heart Association. Circulation. 2016;133(8):802-20.
13. Engels EA, Pfeiffer RM, Fraumeni JF et al. Spectrum of cancer risk among US solid organ transplant recipients. Jama. 2011;306(17):1891-901.
14. Krynitz B, Edgren G, Lindelöf B et al. Risk of skin cancer and other malignancies in kidney, liver, heart and lung transplant recipients 1970 to 2008--a Swedish population-based study. Int J Cancer. 2013;132(6):1429-38
15. Harbron RW, Chapple CL, O'Sullivan JJ et al. Cancer incidence among children and young adults who have undergone x-ray guided cardiac catheterization procedures. Eur J Epidemiol. 2018;33(4):393-401.

16. Abalo KD, Malekzadeh-Milani S, Hascoët S et al. Lympho-hematopoietic malignancies risk after exposure to low dose ionizing radiation during cardiac catheterization in childhood. Eur J Epidemiol. 2023;38(7):821-34.

For peer review only - http://bmjopen.bmj.com/site/about/guidelines.xhtml

17. Christman MP, Castro-Zarraga M, Defaria Yeh D, Liberthson RR, Bhatt AB. Adequacy of cancer screening in adult women with congenital heart disease. *ISRN Cardiol*. 2013;2013:827696.

18. Bergh N, Skoglund K, Fedchenko M et al. Risk of Heart Failure in Congenital Heart Disease: A Nationwide Register-Based Cohort Study. Circulation. 2023;147(12):982-4.

19. Diller GP, Kempny A, Alonso-Gonzalez R, et al. Survival prospects and circumstances of death in contemporary adult congenital heart disease patients under follow-up at a large tertiary centre. *Circulation*. 2015;132(22):2118-25.

20. Venkatesh P, Yan KL, Bravo-Jaimes K, Yang EH, Lluri G. Outcomes of malignancy in adults with congenital heart disease: a single center experience. *Cardiooncology*. 2022;8(1):20.

21. Botto LD, Lin AE, Riehle-Colarusso T, Malik S, Correa A. Seeking causes: Classifying and evaluating congenital heart defects in etiologic studies. *Birth Defects Res A Clin Mol Teratol*. 2007;79(10):714-27.

22. Liu S, Joseph KS, Lisonkova S, et al. Association between maternal chronic conditions and congenital heart defects: a population-based cohort study. *Circulation*. 2013;128(6):583-9.

23. Lee YS, Chen YT, Jeng MJ, et al. The risk of cancer in patients with congenital heart disease: a nationwide population-based cohort study in Taiwan. *PLoS One*. 2015;10(2):e0116844.

24. Ohuchi H, Hayama Y, Nakajima K, Kurosaki K, Shiraishi I, Nakai M. Incidence, predictors, and mortality in patients with liver cancer after Fontan operation. *J Am Heart Assoc*. 2021;10(4):e016617.

25. Goldstein SA, D'Ottavio A, Spears T, et al. Causes of death and cardiovascular comorbidities in adults with congenital heart disease. *J Am Heart Assoc*. 2020;9(14):e016400.

26. Van Bulck L, Goossens E, Morin L, et al. Last year of life of adults with congenital heart diseases: causes of death and patterns of care. *Eur Heart J*. 2022;43(42):4483-92.

27. Williams JL, Torok RD, D'Ottavio A, et al. Causes of death in infants and children with congenital heart disease. *Pediatr Cardiol*. 2021;42(6):1308-15.

28. Dellborg M, Giang KW, Eriksson P, et al. Adults with congenital heart disease: Trends in event-free survival past middle age. *Circulation*. 2023;147(12):930-8.

29. Attarbaschi A, Carraro E, Abla O, et al. Non-Hodgkin lymphoma and pre-existing conditions: spectrum, clinical characteristics and outcome in 213 children and adolescents. *Haematologica*. 2016;101(12):1581-91.

For peer review only - http://bmjopen.bmj.com/site/about/guidelines.xhtml

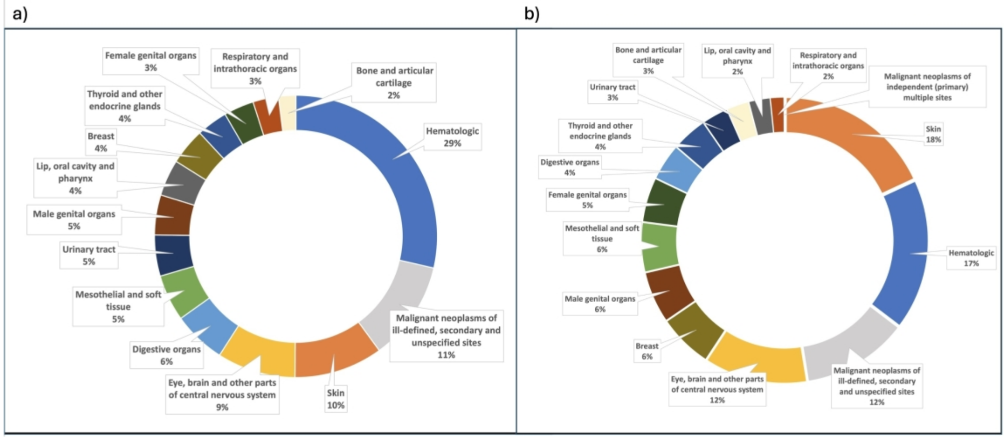

Figure 1 - Cancer diagnoses in a) CHD patients and b) controls without CHD

230x100mm (300 x 300 DPI)

Supplementary tables and figures

Supplementary Table 1. Congenital heart disease diagnosis according to the International Statistical Classification of Diseases and Related Health Problems

Diagnosis	ICD-8	ICD-9*	ICD-10
Common arterial trunk	746,09	745A	Q200
Transposition of the great arteries	746,19	745B	Q203
Tetralogy of Fallot	746,29	745C	Q213
Ventricular septal defect	746,39	745E	Q210
Atrial septal defect/patent foramen ovale	746,42	745F	Q211
Congenital tricuspid stenosis or atresia	746,54	746B	Q224
Ebstein's anomaly	746,54	746C	Q225
Congenital stenosis of the aortic valve	746,73	746D	Q230
Congenital insufficiency of the aortic valve	746,79	746E	Q231
Congenital mitral stenosis	746,59	746F	Q232
Congenital mitral insufficiency	746,59	746G	Q233
Hypoplastic left heart syndrome	746,74	746H	Q234
Congenital subaortic stenosis	746,79	746W	Q244
Cor triatriatum	746,89	746W	Q242
Infundibular pulmonic stenosis	746,63	746W	Q243
Congenital coronary artery anomalies	747,69	746W	Q245
Congenital heart block	746,89	746W	Q246
Coarctation of the aorta	747,19	747B	Q251
Interruption of the aortic arch	747,19	747B	Q252<br>Q253
Other unspecified congenital malformations of the aorta	747,29	747C	Q254<br>Q258<br>Q259
Congenital malformations of the pulmonary artery	747,34<br>747,39	747D	Q255<br>Q256<br>Q257
Congenital malformations of the great veins	747,49<br>747,59	747E	Q260<br>Q261<br>Q262<br>Q263<br>Q264
Cor biloculare	746,89	745H	Q208
Double outlet right ventricle	746,19	745B	Q201
Double outlet left ventricle	746,19	745B	Q202
Double inlet ventricle	746,37	745D	Q204
Congenitally corrected transposition/discordant atrioventricular and ventriculoatrial connection	746,19	745B	Q205

For peer review only

Isomerism of atrial appendages	746,89	745W	Q206
Unspecified congenital malformations of the cardiac chambers	746,89	746X	Q208<br>Q209
Atrioventricular septal defect	746,47<br>746,46<br>746,43	745G	Q212
Aortopulmonary septum defect	746,09	745A	Q214
Other congenital malformations of the cardiac septum	746,89	745W	Q218
Unspecified congenital malformations of the cardiac septum	746,99	745X	Q219
Pulmonary valve atresia	746,64	746A	Q220
Congenital stenosis of the pulmonary valve	746,63	746A	Q221
Congenital pulmonary valve insufficiency	746,69	746A	Q222
Other congenital malformations of the pulmonary valve	746,69	746A	Q223
Hypoplastic right heart syndrome	746,69	746B	Q226
Other congenital malformations of the tricuspid valve	746,54	746B	Q228<br>Q229
Other congenital malformations of aortic and mitral valves	746,89	746W	Q238<br>Q239
Other specified congenital malformations of the heart	746,89	746W	Q248
Unspecified congenital malformations of the heart	746,99	746X	Q249
Patent ductus arteriosus	747,09	747A	Q250

ICD, International Classification of Diseases. *Swedish version of the ICD-9.

For peer review only

Supplementary Table 2. Classification of CHD

		CHD diagnosis	ICD-8	ICD-9	ICD-10
**Complex lesions**	**Lesion group 1:** conotruncal defects	Common arterial trunk	746.09	745A	Q200
		Aortopulmonary septum defect	746.09	745A	Q214
		Double outlet right ventricle	746.19	745B	Q201
		Double outlet left ventricle	746.19	745B	Q202
		Transposition of great vessels	746.19	745B	Q203
		Discordant atrioventricular connection (ccTGA)		745B	Q205
		Tetralogy of Fallot	746.29	745C	Q213
	**Lesion group 2:** severe nonconotruncal defects	Endocardial cushion defects	746.43 746.46 746.47	745G	Q212
		Common ventricle	746.37	745D	Q204
		Hypoplastic left heart syndrome	746.74	745H	Q234
**Non-complex lesions**	**Lesion group 3:** coarcatio of the aorta	Coarctation of the aorta	747.19	747B	Q251
	**Lesion group 4:** Ventricular septal defect	Ventricular septal defect	746.39	745E	Q210
	**Lesion group 5:** Atrial septal defect	Atrial septal defect	746.42	745F	Q211
	**Lesion group 6:** Other heart and circulatory system anomalies	All other congenital heart disease diagnoses that are not included in the above five lesion groups			

Supplementary Table 3. Cancer diagnosis according to the International Statistical Classification of Diseases and Related Health Problems

Diagnosis	ICD-8	ICD-9*	ICD-10
Malignant neoplasms of lip, oral cavity and pharynx	140-149	140-149	C00-C14
Malignant neoplasms of digestive organs	150-159	150-159	C15-C26
Malignant neoplasms of respiratory and intrathoracic organs	160-163	160-165	C30-C39
Malignant neoplasms of bone and articular cartilage	170-171	170	C40-C41
Melanoma and other malignant neoplasms of skin	172-173	172-173	C43-C44
Malignant neoplasms of mesothelial and soft tissue	-	158,162,163, 171, 173	C45-C49
Malignant neoplasms of breast	174	174-175	C50
Malignant neoplasms of female genital organs	180-184	179-184,236C	C51-C58
Malignant neoplasms of male genital organs	185-187	185-187	C60-C63
Malignant neoplasms of urinary tract	188-189	188-189	C64-C68
Malignant neoplasms of eye, brain and other parts of central nervous system	190-192	190-192	C69-C72
Malignant neoplasms of thyroid and other endocrine glands	193-194	193-194	C73-C75
Malignant neoplasms o ill-defined, secondary and unspecified sites	195-198	195-199	C76-C80
Malignant neoplasms, stated or presumed to be primary, of lymphoid, haematopoietic and related tissue	200-209	200-209	C81-C96
Malignant neoplasms of independent (primary) multiple sites	199	199	C97

ICD, International Classification of Diseases. *Swedish version of the ICD-9.

For peer review only

Supplementary Table 4. Syndromes diagnosis according to the International Statistical Classification of Diseases and Related Health Problems

Diagnosis	ICD8	ICD9	ICD10
Downs syndrom	7593	758A	Q90
Noonan	-	-	Q87.1
Di Georges syndrome (22q11-deletion)	-	279L	D82.1
Klinefelter	759,51	758H	Q98
Turners	759,50	758G	Q96
Williams-Buren	-	-	Q93.8
Edwards (trisomi 18)	759,40	758C	Q91
Patau (trisomi 13)	759,41	758B	Q91
CFC (cardio-facio-cutant syndrom, costellossyndrom, CHARGE, Smith-Magenis	-	-	Q87.8
Neurofibromatosis type 1	743,40	237H	Q85.0
Constitutional aplastic anemia (incl Fanconis anemia)	284	284A	D61.0
Heterotaxia (situs inversus)	759.0	759D	Q89.3

Supplementary table 5. Comorbidity diagnoses according to the International Statistical Classification of Diseases and Related Health Problems

Diagnosis	ICD8	ICD9	ICD10
Hypertension	400-404	401-405	I10-I15
Diabetes mellitus	250	250	E10-E14
Heart failure	427.00	428	I50
Myocardial infarction	410	410	I21
Atrial fibrillation	427.92	427D	I48
Ischemic stroke	433, 434	434, 436	I63-I64

Supplementary table 6. Follow-up time

Follow-up time	CHD	Controls
All cancer patients		
Mean, years (SD)	**9.2±9.3***	**7.8±7.8***
Median, years (IQR)	6.3 (1.9-14.5)	5.3 (2.0-11.3)
Patients with cancer who died		
Mean, years (SD)	3.3±6.6	2.9±4.9
Median, years (IQR)	**0.8 (0.2-3.3)***	**1.3 (0.7-3.0)***
Patients with cancer who died (excl. syndromes and transplant recipients)		
Mean, years (SD)	3.7±7.1	2.8±4.6
Median, years (IQR)	1.0 (0.3-3.6)	1.2 (0.7-2.9)

****Numbers in bold denotes significance (p <0.05)***

For peer review only

Supplementary table 7. Cancer diagnosis in patients with CHD and controls who died (all patients)

Cancer diagnosis	Cases	Controls
Malignant neoplasms, stated or presumed to be primary, of lymphoid, haematopoietic and related tissue	59 (28.5%)	117 (15.4%)
Malignant neoplasms of ill-defined, secondary and unspecified sites	43 (20.8%)	198 (26.0%)
Malignant neoplasms of eye, brain and other parts of central nervous system	26 (12.6%)	147 (19.3%)
Malignant neoplasms of digestive organs	17 (8.2%)	46 (6.0%)
Malignant neoplasms of mesothelial and soft tissue	13 (6.3%)	59 (7.7%)
Malignant neoplasms of urinary tract	<10	20 (2.6%)
Malignant neoplasms of thyroid and other endocrine glands	<10	30 (3.9%)
Malignant neoplasms of lip, oral cavity and pharynx	<10	11 (1.4%)
Malignant neoplasms of breast	<10	28 (3.7%)
Malignant neoplasms of female genital organs	<10	23 (3.0%)
Malignant neoplasms of respiratory and intrathoracic organs	<10	17 (2.2%)
Malignant neoplasms of bone and articular cartilage	<5	30 (3.9%)
Melanoma and other malignant neoplasms of skin	<5	24 (3.1%)
Malignant neoplasms of male genital organs	<5	10 (1.3%)
Malignant neoplasms of independent (primary) multiple sites	0	<5

Supplementary table 8. Cancer diagnosis in patients with CHD and controls who died (excluding syndromes and transplant recipients)

Cancer diagnosis	Cases	Controls
Malignant neoplasms of ill-defined, secondary and unspecified sites	40 (27.0%)	193 (26.7%)
Malignant neoplasms of eye, brain and other parts of central nervous system	22 (14.9%)	141 (19.4%)
Malignant neoplasms, stated or presumed to be primary, of lymphoid, haematopoietic and related tissue	21 (14.2%)	109 (15.0%)
Malignant neoplasms of digestive organs	12 (8.1%)	45 (6.2%)
Malignant neoplasms of mesothelial and soft tissue	12 (8.1%)	51 (7.0%)
Malignant neoplasms of thyroid and other endocrine glands	<10	29 (4.0%)
Malignant neoplasms of breast	<10	27 (3.7%)
Malignant neoplasms of female genital organs	<10	23 (3.2%)
Malignant neoplasms of lip, oral cavity and pharynx	<10	11 (1.5%)
Malignant neoplasms of respiratory and intrathoracic organs	<10	16 (2.2%)
Malignant neoplasms of urinary tract	<5	20 (2.8%)
Malignant neoplasms of bone and articular cartilage	<5	29 (4.0%)
Malignant neoplasms of male genital organs	<5	<10
Melanoma and other malignant neoplasms of skin	<5	20 (2.8%)
Malignant neoplasms of independent (primary) multiple sites	0	<5

For peer review only

Supplementary table 9. Deaths

Deaths	CHD	Controls
Female/Male	69/70	213/214
Birth period (patients with cancer)		
- 1970-1979	41	183
- 1980-1989	36	89
- 1990-1999	34	82
- 2000-2009	14	60
- 2010-2017	14	13
Lesion group (patients with cancer)		
- Complex lesions	35 (29.2%)	
- Non-complex lesions	104 (16.3%)	

Supplementary table 10. Mortality IR divided by gender, lesion groups and birth cohorts

Mortality IR*	CHD (incl/excl syndromes+Tx)	Controls (incl/excl syndromes+Tx)
In total cancer population	1.99 / 1.72	1.49 / 1.46
Cancer population divided by:		
- ***Gender***		
Male	2.03 / 1.82	1.56 / 1.51
Female	1.95 / 1.61	1.43 / 1.41
- ***Lesion groups***		
Complex lesions	3.70 / 2.40	
Non-complex lesions	1.72 / 1.63	
- ***Birth period***		
1970-1979	1.89 / 1.78	1.46 / 1.41
1980-1989	2.05 / 1.50	1.24 / 1.19
1990-1999	1.78 / 1.43	1.60 / 1.63
2000-2009	1.52 / 1.23	1.86 / 1.90
2010-2017	6.13 / 6.88	2.54 / 2.01

* per 100 person-years

Supplementary table 11. Mortality HR divided by gender, lesion groups and birth cohorts

Mortality hazard ratio	HR (95% CI)	
Divided by gender	Incl. syndromes and tpx	Excl. syndromes and tpx
Unadjusted model	Gender 1 **1.48 (1.13-1.93)**<br>Gender 2 **1.62 (1.23-2.13)**	Gender 1 **1.36 (1.00-1.87)**<br>Gender 2 1.33 (0.95-1.86)
Model 1 (adjusted for birth period and age at cancer diagnosis)	Gender 1 **1.36 (1.04-1.79)**<br>Gender 2 **1.36 (1.03-1.80)**	Gender 1 1.27 (0.92-1.74)<br>Gender 2 1.14 (0.81-1.60)
Model 2 (adjusted for birth period, age at cancer diagnosis and comorbidities)	Gender 1 1.32 (0.99-1.74)<br>Gender 2 1.18 (0.88-1.59)	Gender 1 1.24 (0.90-1.72)<br>Gender 2 1.09 (0.77-1.54)
Divided by lesion groups	Incl. syndromes and tpx	Excl. syndromes and tpx
Unadjusted model	Complex **2.96 (1.97-4.46)**<br>Non-complex **1.33 (1.07-1.65)**	Complex **1.99 (1.12-3.55)**<br>Non-complex 1.27 (0.99-1.64)
Model 1 (adjusted for birth period, age at cancer diagnosis and gender)	Complex **2.64 (1.72-4.07)**<br>Non-complex **1.17 (0.94-1.46)**	Complex 1.76 (0.98-3.17)<br>Non-complex 1.14 (0.89-1.47)
Model 2 (adjusted for birth period, age at cancer diagnosis, gender and comorbidities)	Complex **2.32 (1.45-3.72)**<br>Non-complex 1.11 (0.88-1.39)	Complex 1.70 (0.90-3.19)<br>Non-complex 1.11 (0.86-1.44)
Divided by birth cohorts	Incl. syndromes and tpx	Excl. syndromes and tpx
Unadjusted model	1970-1989 **1.71 (1.33-2.21)**<br>1990-2017 **1.36 (1.01-1.83)**	1970-1989 **1.49 (1.11-2.00)**<br>1990-2017 1.16 (0.80-1.68)
Model 1 (adjusted for age at cancer diagnosis and gender)	1970-1989 **1.50 (1.16-1.94)**<br>1990-2017 1.16 (0.86-1.57)	1970-1989 1.32 (0.98-1.77)<br>1990-2017 1.01 (0.70-1.47)
Model 2 (adjusted for age at cancer diagnosis, gender and comorbidities)	1970-1989 **1.37 (1.05-1.80)**<br>1990-2017 1.08 (0.80-1.48)	1970-1989 1.26 (0.93-1.71)<br>1990-2017 1.00 (0.69-1.46)

For peer review only

Supplementary table 12. Age at cancer diagnosis

All patients	CHD	Controls
Age 0-17 years at cancer diagnosis, n (%)	451 (59.5%)	1416 (38.6%)
Age 18+ years at cancer diagnosis, n (%)	307 (40.5%)	2254 (61.4%)
Mean, years (SD)	**16.5±14.5***	**22.7±14.2***
Median, years (IQR)	**13.0 (2.9–30.0)***	**24.6 (8.6–35.1)***
**All patients – excl. syndromes/Tx**	**CHD**	**Controls**
Age 0-17 years at cancer diagnosis, n (%)	299 (53.0%)	1345 (37.6%)
Age 18+ years at cancer diagnosis, n (%)	265 (47.0%)	2229 (62.4%)
Mean, years (SD)	**18.5±14.8***	**23.0±14.1***
Median, years (IQR)	**16.6 (3.9-32.2)**	**25.0 (9.1-35.2)***
**Patients who died**	**CHD**	**Controls**
Mean, years (SD)	**14.2±14.1***	**17.0±13.5***
Median, years (IQR)	**9.3 (1.7-24.0)***	**14.8 (3.8-29)***
**Patients who died – excl. syndromes/Tx**	**CHD**	**Controls**
Mean, years (SD)	16.2±15.0	17.2±13.6
Median, years (IQR)	13.5 (1.6-29.9)	15.1 (3.9-29.1)

CHD=congenital heart disease. Tx= transplant recipients. SD=standard deviation. IQR=interquartile range.
Numbers in bold denotes significance (p <0.05)

Supplementary table 13. Age at death in patients with cancer

Age at death – all patients	CHD	Controls
Mean, years (SD)	17.5±14.8	19.8±13.9
Median, years (IQR)	**15.1 (3.6-30.7)***	**18.5 (6.1-32.7)***
**Age at death – excl. syndromes/Tx**	**CHD**	**Controls**
Mean, years (SD)	19.8±15.5	20.0±13.9
Median, years (IQR)	19.5 (4.7-35.6)	18.7 (6.1-32.7)

CHD=congenital heart disease. Tx= transplant recipients. SD=standard deviation. IQR=interquartile range.
Numbers in bold denotes significance (p <0.05)

Supplementary figure 1. Kaplan-Meier survival

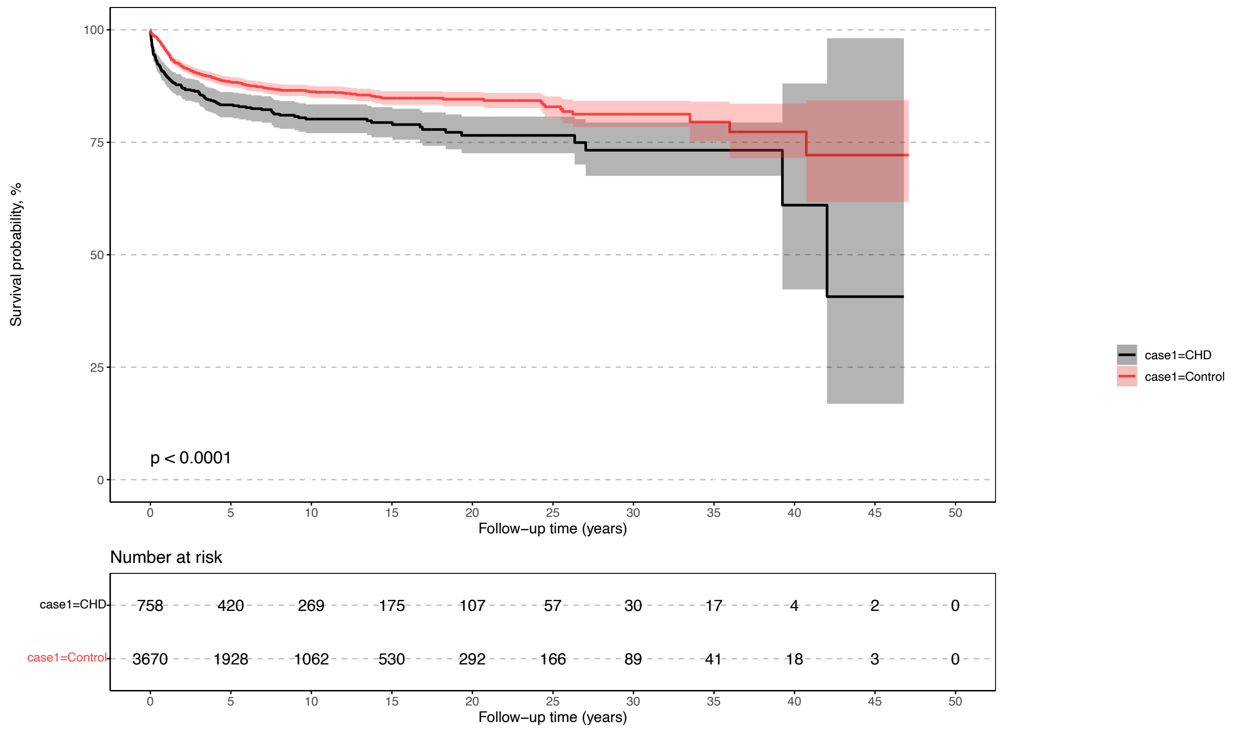

For peer review

Supplementary figure 2a. Cause of death patients with CHD and cancer: complex lesions

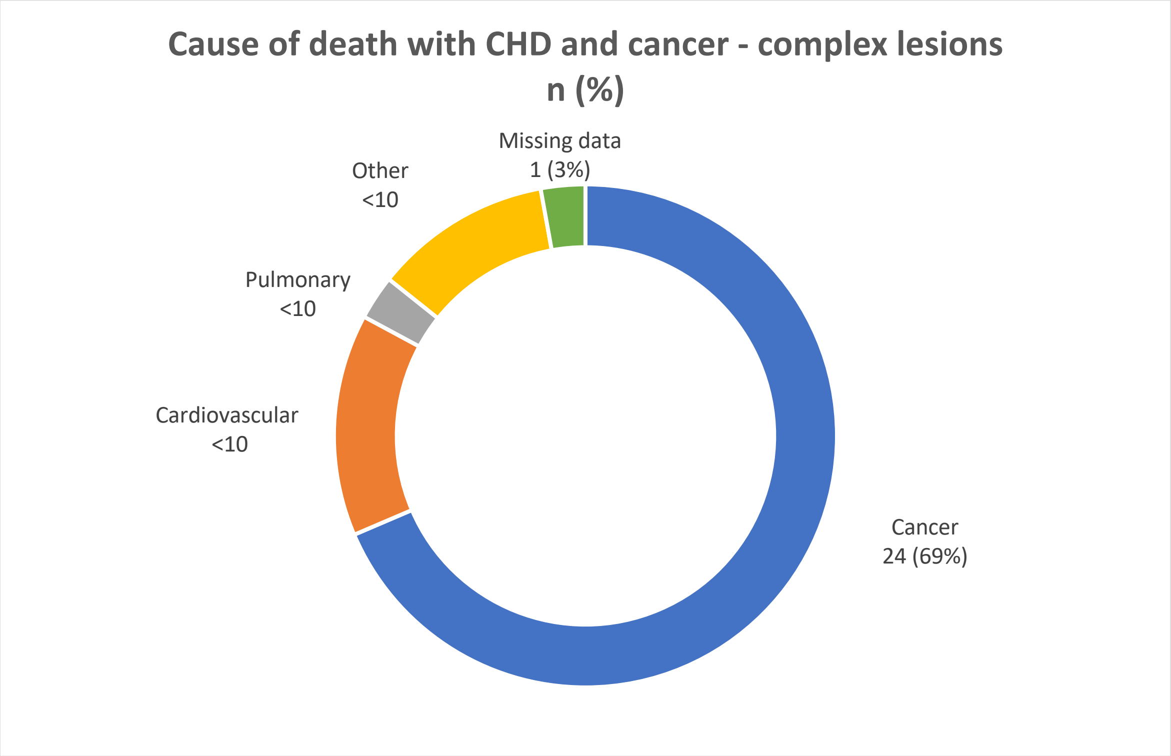

* All cause of deaths from patients and controls from the year 2017 were missing, because of later registration.

Supplementary figure 2b. Cause of death patients with CHD and cancer: non-complex lesions

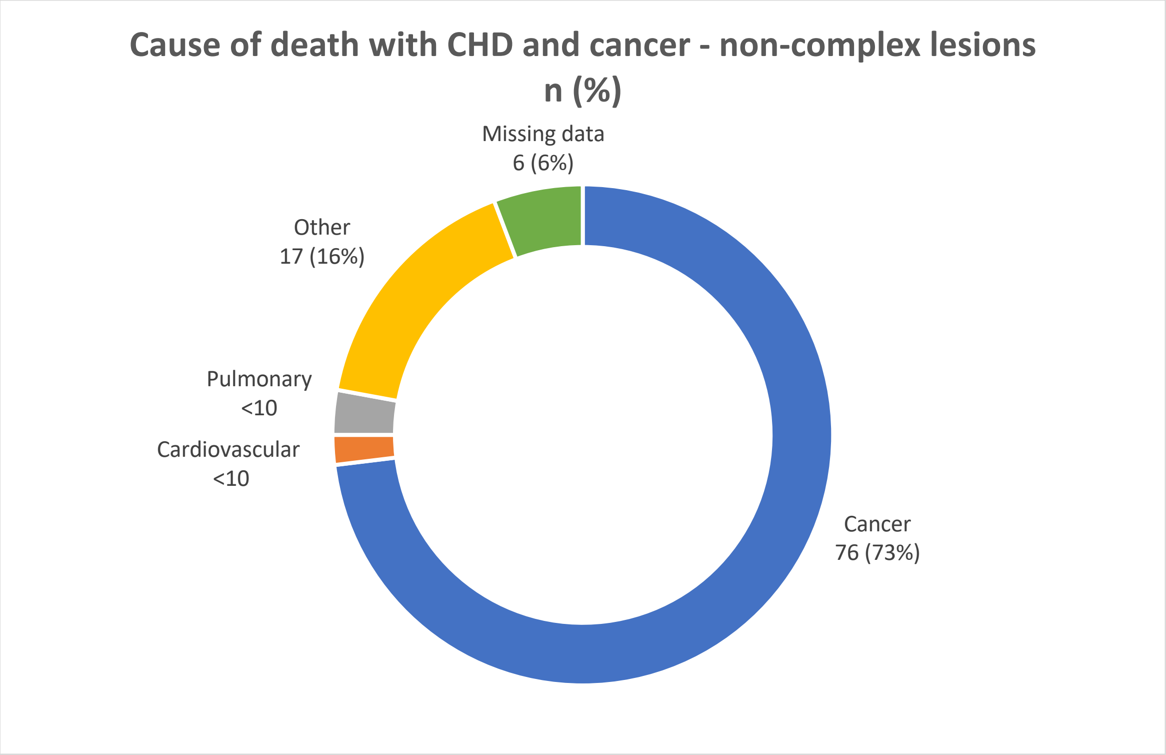

* All cause of deaths from patients and controls from the year 2017 were missing, because of later registration.

Supplementary figure 3a. Age at death in patients with CHD and cancer cause-of death (all patients)

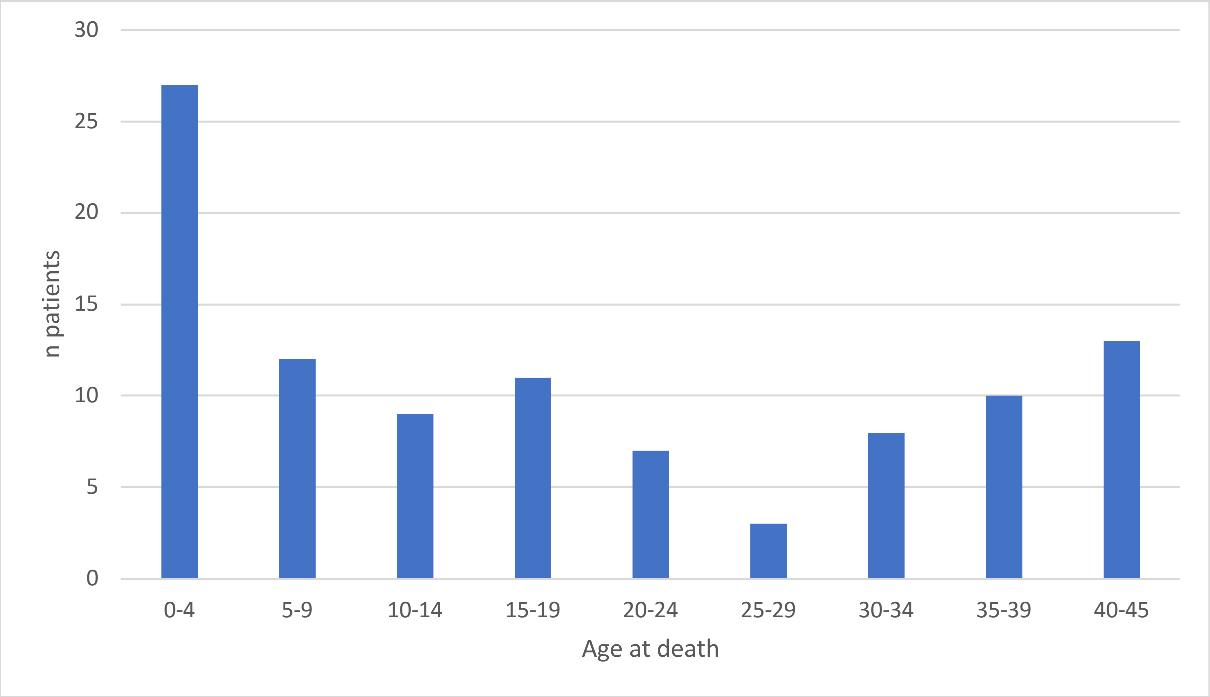

Supplementary figure 3b. Age at death in patients with CHD and cancer cause-of death (excl. syndromes and transplant recipients)

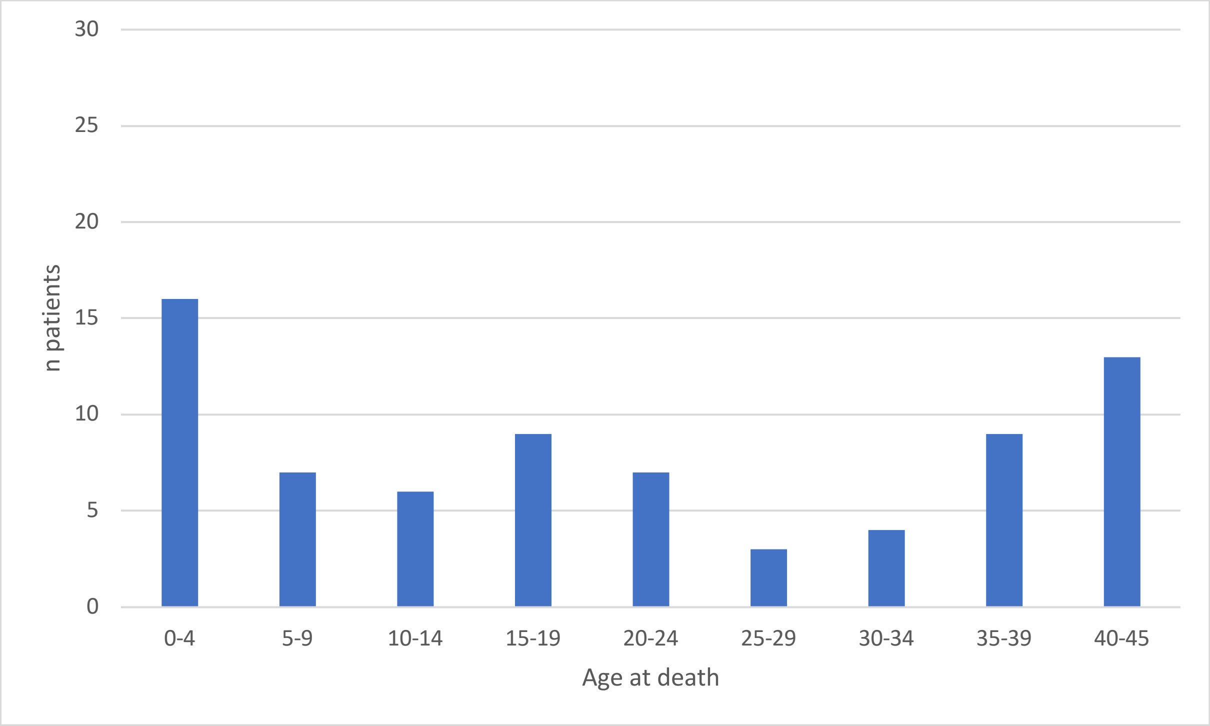

STROBE Statement—Checklist of items that should be included in reports of ***cohort studies***

	Item No	Recommendation
**Title and abstract**	1	(*a*) Indicate the study's design with a commonly used term in the title or the abstract - page 2
		(*b*) Provide in the abstract an informative and balanced summary of what was done and what was found - page 2
**Introduction**		
Background/rationale	2	Explain the scientific background and rationale for the investigation being reported - page 3
Objectives	3	State specific objectives, including any prespecified hypotheses - page 3-4
**Methods**		
Study design	4	Present key elements of study design early in the paper - page 5
Setting	5	Describe the setting, locations, and relevant dates, including periods of recruitment, exposure, follow-up, and data collection - page 5-6
Participants	6	(*a*) Give the eligibility criteria, and the sources and methods of selection of participants. Describe methods of follow-up - page 6-7
		(*b*) For matched studies, give matching criteria and number of exposed and unexposed - page 5
Variables	7	Clearly define all outcomes, exposures, predictors, potential confounders, and effect modifiers. Give diagnostic criteria, if applicable - page 6-7
Data sources/ measurement	8*	For each variable of interest, give sources of data and details of methods of assessment (measurement). Describe comparability of assessment methods if there is more than one group - page 6-7
Bias	9	Describe any efforts to address potential sources of bias - page 14
Study size	10	Explain how the study size was arrived at - page 5
Quantitative variables	11	Explain how quantitative variables were handled in the analyses. If applicable, describe which groupings were chosen and why - page 6
Statistical methods	12	(*a*) Describe all statistical methods, including those used to control for confounding - page 6-7
		(*b*) Describe any methods used to examine subgroups and interactions - page 6-7
		(*c*) Explain how missing data were addressed - page 9
		(*d*) If applicable, explain how loss to follow-up was addressed - N/A
		(*e*) Describe any sensitivity analyses - N/A
**Results**		
Participants	13*	(a) Report numbers of individuals at each stage of study—eg numbers potentially eligible, examined for eligibility, confirmed eligible, included in the study, completing follow-up, and analysed - page 7
		(b) Give reasons for non-participation at each stage - N/A
		(c) Consider use of a flow diagram -
Descriptive data	14*	(a) Give characteristics of study participants (eg demographic, clinical, social) and information on exposures and potential confounders - page 7 and table 1
		(b) Indicate number of participants with missing data for each variable of interest- page 9
		(c) Summarise follow-up time (eg, average and total amount) - page 7 and supplementary table 6
Outcome data	15*	Report numbers of outcome events or summary measures over time - page 8-10
Main results	16	(*a*) Give unadjusted estimates and, if applicable, confounder-adjusted estimates and their precision (eg, 95% confidence interval). Make clear which confounders were adjusted for and why they were included - page 8-10
		(*b*) Report category boundaries when continuous variables were categorized - page 9-10
		(*c*) If relevant, consider translating estimates of relative risk into absolute risk for a meaningful time period -

For peer review only

For peer review only - http://bmjopen.bmj.com/site/about/guidelines.xhtml

Other analyses	17	Report other analyses done—eg analyses of subgroups and interactions, and sensitivity analyses - page 8-10 (exclusion of patients with syndromes and transplant recipients)
**Discussion**		
Key results	18	Summarise key results with reference to study objectives - page 10-13
Limitations	19	Discuss limitations of the study, taking into account sources of potential bias or imprecision. Discuss both direction and magnitude of any potential bias - page 14
Interpretation	20	Give a cautious overall interpretation of results considering objectives, limitations, multiplicity of analyses, results from similar studies, and other relevant evidence - page 15
Generalisability	21	Discuss the generalisability (external validity) of the study results - page 14
**Other information**		
Funding	22	Give the source of funding and the role of the funders for the present study and, if applicable, for the original study on which the present article is based - page 15

*Give information separately for exposed and unexposed groups.

Note: An Explanation and Elaboration article discusses each checklist item and gives methodological background and published examples of transparent reporting. The STROBE checklist is best used in conjunction with this article (freely available on the Web sites of PLoS Medicine at http://www.plosmedicine.org/, Annals of Internal Medicine at http://www.annals.org/, and Epidemiology at http://www.epidem.com/). Information on the STROBE Initiative is available at http://www.strobe-statement.org.

For peer review only
